# Black Carbon as an Additional Indicator of the Adverse Health Effects of Airborne Particles Compared with PM_10_ and PM_2.5_

**DOI:** 10.1289/ehp.1003369

**Published:** 2011-08-02

**Authors:** Nicole A.H. Janssen, Gerard Hoek, Milena Simic-Lawson, Paul Fischer, Leendert van Bree, Harry ten Brink, Menno Keuken, Richard W. Atkinson, H. Ross Anderson, Bert Brunekreef, Flemming R. Cassee

**Affiliations:** 1 National Institute for Public Health and the Environment, Bilthoven, the Netherlands; 2 Institute for Risk Assessment Sciences, Utrecht University, Utrecht, the Netherlands; 3 Division of Population Health Sciences and Education and the Medical Research Council–Health Protection Agency Centre for Environment and Health, St. George’s, University of London, London, United Kingdom; 4 Netherlands Environmental Assessment Agency, Bilthoven, the Netherlands; 5 Energy Research Center of the Netherlands, Petten, the Netherlands; 6 Netherlands Applied Research Organization, Utrecht, the Netherlands; 7 Medical Research Council–Health Protection Agency for Environment and Health, King’s College, London, United Kingdom; 8 Julius Center for Health Sciences and Primary Care, University Medical Center Utrecht, Utrecht, the Netherlands

**Keywords:** air quality management, black carbon, combustion particles, health effects, particulate matter, review

## Abstract

Background: Current air quality standards for particulate matter (PM) use the PM mass concentration [PM with aerodynamic diameters ≤ 10 μm (PM_10_) or ≤ 2.5 μm (PM_2.5_)] as a metric. It has been suggested that particles from combustion sources are more relevant to human health than are particles from other sources, but the impact of policies directed at reducing PM from combustion processes is usually relatively small when effects are estimated for a reduction in the total mass concentration.

Objectives: We evaluated the value of black carbon particles (BCP) as an additional indicator in air quality management.

Methods: We performed a systematic review and meta-analysis of health effects of BCP compared with PM mass based on data from time-series studies and cohort studies that measured both exposures. We compared the potential health benefits of a hypothetical traffic abatement measure, using near-roadway concentration increments of BCP and PM_2.5_ based on data from prior studies.

Results: Estimated health effects of a 1-μg/m^3^ increase in exposure were greater for BCP than for PM_10_ or PM_2.5_, but estimated effects of an interquartile range increase were similar. Two-pollutant models in time-series studies suggested that the effect of BCP was more robust than the effect of PM mass. The estimated increase in life expectancy associated with a hypothetical traffic abatement measure was four to nine times higher when expressed in BCP compared with an equivalent change in PM_2.5_ mass.

Conclusion: BCP is a valuable additional air quality indicator to evaluate the health risks of air quality dominated by primary combustion particles.

Particulate matter (PM) is a heterogeneous mixture varying in physicochemical properties depending on meteorological conditions and emission sources [World Health Organization (WHO) 2006]. Current air quality standards for PM use the mass concentration of PM [PM with aerodynamic diameters ≤ 10 μm (PM_10_) or ≤ 2.5 μm (PM_2.5_)] as a metric, supported by health studies showing robust associations between ambient PM mass concentrations and a wide array of adverse health effects (WHO 2006). However, it is likely that not every PM component is equally important in causing these health effects (WHO 2007).

Combustion-related particles are thought to be more harmful to health than PM that is not generated by combustion (Krzyzanowski et al. 2005; WHO 2007). In urban areas, road traffic is a major source of combustion PM [Health Effects Institute (HEI) 2010]. In a systematic review of the literature, Krzyzanowski et al. (2005) concluded that transport-related air pollution contributes to an increased risk of death, particularly from cardiopulmonary causes, and that it increases the risk of respiratory symptoms and diseases that are not related to allergies. In a more recent review of traffic-related air pollution, HEI (2010) concluded that there was sufficient evidence to support a causal relationship between exposure to traffic-related air pollution and exacerbation of asthma, and suggestive evidence of a causal relationship with onset of childhood asthma, nonasthma respiratory symptoms, impaired lung function, total and cardiovascular mortality, and cardiovascular morbidity.

Combustion particles also derive from a variety of sources other than motorized road traffic, including wood and coal burning, shipping, and industrial sources, and these sources may contribute significantly to ambient combustion particle concentrations (WHO 2006). There is increasing concern that current mass-based PM standards are not well suited for characterizing health risks of air pollution near sources of combustion particles, such as motorized traffic on major roads or in wood-smoke–dominated communities. Furthermore, emission reduction measures such as the use of particle traps or the introduction of environmental zones are thought to be effective in reducing exposure to traffic-related air pollution, but the estimated impact of such measures is relatively small when expressed in relation to a reduction in the PM mass concentration (Lefebvre et al. 2011; Millstein and Harley 2010; Tonne et al. 2008). Nitrogen dioxide (NO_2_) is a regulated component of air pollution that is also used as an indicator of traffic-related air pollution in health impact assessment and air quality management (Tonne et al. 2008). However, NO_2_ is not a suitable indicator to evaluate the effect of traffic abatement measures on exposure to combustion particles because some abatement measures, such as filters on diesel fueled vehicles, may increase NO_2_ levels (Millstein and Harley 2010). In addition, spatial gradients near roadways are less pronounced for NO_2_ than for black smoke (BS) and particle number because of high background concentrations of NO_2_ (Krzyzanowski et al. 2005). This is less of a concern for nitric oxide (NO) and nitrogen oxides (NO_x_), which is the sum of NO and NO_2_, but these components are not regulated currently, and they do not appear to be toxicologically important at current ambient levels.

These considerations led us to consider whether another PM metric might better reflect the health effects of combustion-related air pollution than PM mass or provide an additional indicator of the effectiveness of air quality management plans aimed at reducing exposure to particles from combustion sources. We have deliberately used the term “additional indicator” because we do not claim that all health effects associated with PM mass in previous studies can be attributed to a marker of combustion particles.

Possible candidates for such an indicator are measures of black carbon particles [BCP; e.g., BS, black carbon (BC), absorption coefficient (Abs), and elemental carbon (EC)] and organic carbon (OC), particle number concentration, particle surface area, and combustion-specific PM components. The extent of available data to support the health relevance of these measures varies widely; most of the information is available for BS. Evidence is also available from both toxicological and epidemiological studies of health effects of ultrafine PM (Knol et al. 2009), but the costs and complexity of monitoring and concerns about the validity of central-site monitoring to estimate personal exposure to ultrafine particles, which is characterized by particle number concentrations rather than particle mass, probably limit the feasibility of particle number as an additional metric.

A WHO (2003) working group recommended reevaluating BS as an indicator of traffic-related air pollution, but a systematic comparison using PM versus BCP indicators to estimate health effects is still lacking. Grahame and Schlesinger (2010) reviewed the evidence of effects of BC on cardiovascular health end points and concluded that it may be desirable to promulgate a BC PM_2.5_ standard. However, no systematic comparison with PM_10_ or PM_2.5_ mass was included. Conversely, Smith et al. (2009) noted that although the results of their time-series meta-analysis suggest larger effects per unit mass of sulfate than of BS; this distinction was less clear in the few studies that directly compared estimated effects of both indicators. This observation indicates the need to critically compare studies that have measured PM mass as well as BCP.

BCP would be a useful indicator in addition to particle mass if *a*) health risks associated with BCP are quantitatively or qualitatively different from those associated with PM mass on a mass unit basis, *b*) the spatial contrast related to the vicinity of combustion sources and the impact of emission reductions is larger than for PM mass, and *c*) BCP and particle mass are not—or at least not usually—highly correlated in time or space.

In this article we evaluate the value of BCP as an indicator of the adverse health effects of combustion particles in addition to PM mass. We performed a systematic review and meta-analysis of epidemiological studies that measured both PM mass and BCP and estimated the potential impact on life expectancy of a traffic abatement measure using the pooled concentration–response functions for BCP and PM_2.5_. Although we focused on traffic-related particles, we refer to combustion particles in general because health effects estimates were based on measurements of BCP from all combustion sources, not exclusively traffic.

## Materials and Methods

*Measurement methods for BCP.* BCP as a metric of combustion-derived PM may be determined by optical methods or thermal-optical analysis. Optical methods used to measure BS, BC, and the Abs of PM are all based on the blackness of a filter sample. For BS the amount of reflected light is converted to total PM mass, whereas for BC it is converted to EC mass. Although BS is expressed in micrograms per cubic meter, there is no consistent relationship to PM mass because conversion of the optical measurement to mass units depends on location, season, and type of combustion particles (Hoek et al. 1997). BS has been used in Europe since the 1920s, and although it has been phased out since the introduction of PM_10_ as the new regulatory particulate metric, some countries, including the Netherlands and the United Kingdom, continue to measure BS in selected locations (Bloemen et al. 2007). EC is determined by thermal-optical analysis in a multistep process, typically resulting in a measurement of OC as well. There are several different protocols to measure EC, and results may differ by up to a factor of 2 (HEI 2010). Extreme care is thus necessary when comparing data on EC from different studies. Concentrations for EC averaged over 24 hr are available for regional and urban monitoring sites through the U.S. IMPROVE (Interagency Monitoring of Protected Visual Environments) network and the U.S. Environmental Protection Agency Chemical Speciation Network, but there are no national monitoring networks for EC in Europe. Here we use “BCP” as a generic term for any of the different metrics (BS, EC, BC, or Abs) but refer to the study-specific metric when describing individual studies.

The different optical measurements for BCP (BS, BC, and Abs) are highly correlated (Quincey 2007; Roorda-Knape et al. 1998). Although thermally determined EC and optical measures of BC are also highly correlated, the quantitative relation between them varies between countries, cities, and type of location (e.g., regional, urban, traffic), highlighting the need for site-specific calibrations (Cyrys et al. 2003; Schaap and van der Gon 2007). Differences between EC measurement methods add to this variation. To facilitate comparisons among studies that used different measures of BCP, we derived a BS-to-EC conversion factor based on the average increase in EC associated with a 10-μg/m^3^ increase in BS reported in 11 studies with information on both measures (Adams et al. 2002; Cyrys et al. 2003; Edwards et al. 1983; Erdman et al. 1993; Janssen et al. 2001; Kinney et al. 2000; Lena et al. 2002; Schaap and van der Gon 2007). Based on this analysis, we assume by default that 10 μg/m^3^ BS is equivalent to 1.1 μg/m^3^ EC [Supplemental Material, Table A1 (http://dx.doi.org/10.1289/ehp.1003369)]. In addition, we conducted sensitivity analyses using conversion factors over the range of the estimates from the individual studies (0.5–1.8 μg/m^3^ EC per 10 μg/m^3^ BS).

*Systematic review of health effects of BCP compared with PM mass.* Literature search. We conducted a search for peer-reviewed literature in PubMed (National Library of Medicine 2007) for epidemiological studies that evaluated the health effects of (a measure of) PM mass as well as health effects of (a measure of) BCP. We used the following key words: (British smoke or black smoke or black carbon or elemental carbon or EC or soot or absorbance or absorption coefficient) AND [particles or particulate matter or particulates or particulate air pollution or fine partic* or “PM10” or “PM2.5” or “PM(2.5)” or “PM(10)” or sulfate* or sulphate*] AND (mortality or cohort or hospital or emergency).

We scanned all abstracts and retrieved papers that potentially included effect estimates for PM mass as well as BCP. For acute health effects, we considered only time-series studies on daily mortality and hospital admissions or emergency department visits because these are generally more similar in design and are therefore more likely to allow meta-analyses than are studies on, for example, symptoms or biomarkers. For health effects due to long-term exposure, we considered only cohort studies because they have provided the most relevant data for setting air pollution standards.

For the time-series studies, we also used the Air Pollution Epidemiology Database (APED; St George’s, University of London, London, UK) to identify suitable studies. This database comprises standardized estimates extracted from ecological time-series studies identified by systematic review that meet certain quality criteria, with the last retrieval performed on 15 May 2009 (Smith et al. 2009). We searched APED for estimates related to effects of PM_10_, PM_2.5_, PM with aerodynamic diameter ≤ 13 (PM_13_), total suspended particulates (TSP), or sulfate as well as BS, BC, or EC.

We identified 40 papers on time-series studies on daily mortality or hospital admissions that included area-specific estimates for both PM and BCP, and 17 papers on cohort studies. The APED search identified six papers that were not identified in the PubMed search, but four were excluded because more recent estimates from the same city were available. The search identified four papers published after the last APED systematic review in May 2009.

For the time-series studies on daily mortality and hospital admissions, we excluded five papers on TSP because more recent data, including effect estimates for PM_10_, were available for most of the cities. Also, we excluded one paper on a rare health end point (hospital admissions for headache); a total of 34 papers were included in the review. All these studies had adjusted for major confounders: seasonality and nonlinear function of temperature and relative humidity. For the cohort studies, we excluded two papers on birth outcomes.

Meta-analysis. For the time-series studies, we calculated pooled fixed and random effects relative risk (RR) estimates for all health end points for which estimates from at least three different studies were available for the same age group and for different cities. We report random effect estimates as significant heterogeneity was observed (*p* < 0.05) among individual estimates for some end points. In case of no heterogeneity, fixed and random effect estimates are similar, so we report random effect estimates for all end points for reasons of consistency. If estimates for multiple lags were reported, we used the estimate discussed by the author, as indicated in APED as “selected” lag. If multiple risk estimates were available from the same city, we only included the most recent estimate, and if the study area was part of a larger administrative area included in another paper (e.g., the Netherlands rather than Amsterdam), we included results for the larger area only. Finally, we excluded city-specific estimates for which PM_10_ was partly derived from BS.

We calculated summary fixed and random effects estimates using the metan procedure in STATA (version 11.2; StataCorp, College Station, TX, USA), as described by Harris et al. (2008). In order to calculate pooled estimates and compare estimated effects for BS and PM per mass unit, we converted RRs for BS to RRs for EC using the average conversion factor (10 μg/m^3^ BS = 1.1 μg/m^3^ EC) or the range of conversion factors from individual studies (i.e., 0.5–1.8) for sensitivity analysis.

We expressed pooled effect estimates per 10 μg/m^3^ (for BS and PM_10_) or 1 μg/m^3^ (for PM_2.5_ and EC). To compare effects based on comparable contrasts, we calculated the average ratio of the interquartile ranges (IQRs) for PM mass and BCP and compared it with the ratio of RR–BCP:RR–PM mass. We could not use study-specific IQRs to estimate pooled effects because IQRs were not available for all studies.

*Exposure contrast in BCP compared with PM mass.* We identified studies that simultaneously measured PM mass and BCP concentrations ≤ 50 m from busy roads (as defined as such by the authors) and at background locations and calculated the ratio between these concentrations. To calculate the roadside increment (which we define as the difference between traffic and background concentrations) for PM_2.5_ and EC, we averaged measurements at different traffic locations within the same study area to derive a single value for each study area, and we converted BS and Abs concentrations to EC using the 10 μg/m^3^ BS to 1.1 μg/m^3^ EC conversion factor (or a range of conversion factors) as described above. We then divided the area-specific average difference between traffic and background EC concentrations by the corresponding average difference between traffic and background PM_2.5_ concentrations to estimate the percentage of EC in the roadside increment of PM_2.5_

*Comparison of estimated health benefits of traffic abatement measures using PM_2.5_ or BCP.* To illustrate the potential implications of using BCP as an air quality indicator, we estimated the health benefits of a traffic abatement measure for populations living along busy roads based on both PM_2.5_ mass and BCP. We used the average and 95% confidence interval (CI) of the percentage EC in the roadside increment in PM_2.5_ (calculated as described above) to estimate the health benefits of a hypothetical traffic abatement policy measure resulting in a 1-μg/m^3^ reduction in PM_2.5_ mass. This approach assumes that the reduction in BCP resulting from traffic abatement will be proportional to the decrease in PM mass by the percentage of EC in the roadside increment for PM mass, an assumption that will not hold for all policies.

We estimated the increase in life expectancy that would result from such a traffic abatement policy using life table calculations, as described by Miller and Hurley (2003), for a hypothetical population of 500,000 people 18–64 years of age, distributed in age categories comparable to the 2008 Dutch population. We estimated the effects on this population for a lifetime.

## Results

**Figure 1:**
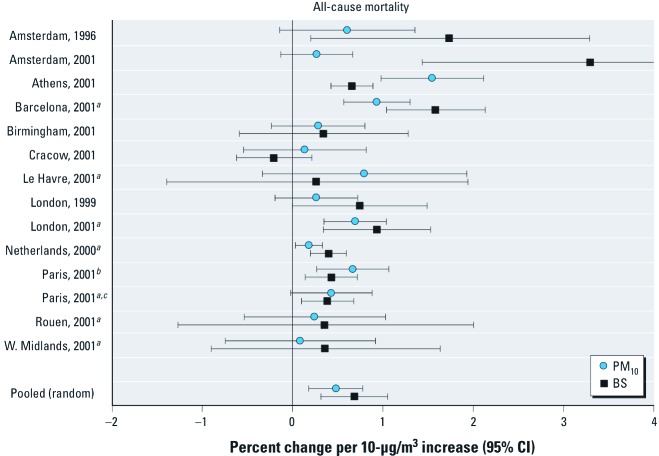
Single-city, single-pollutant estimates for PM_10_ and BS and all-cause mortality. Year indicates year of publication. References: Amsterdam, 1996 [Verhoeff et al. (1996)]; Amsterdam, 2001 [Roemer and van Wijnen (2001a)]; Athens, Barcelona, Birmingham, Cracow, London, and Paris, 2001 [Katsouyanni et al. (2001)]; Le Havre, Paris, and Rouen, 2001 [Zeghnoun et al. (2001)]; London, 1999 [Bremner et al. (1999)]; the Netherlands, 2000 [Hoek et al. (2000)]; West Midlands, 2001 [Anderson et al. (2001)]. ***^a^***Cities included in the pooled estimate. ***^b^***Zeghnoun et al. (2001). ***^c^***Katsouyanni et al. (2001).

*Health effects of BCP compared with PM mass.* Studies on BCP and PM_10_. Most papers concerned time-series studies on PM_10_ and BS (as a measure of BCP) conducted in Europe. We present random effects estimates for the percent change in each outcome with a 10-μg/m^3^ increase in PM_10_ or BS in Table 1. Information and effect estimates for all individual studies, and tests of heterogeneity and fixed effects estimates for studies included in meta-analyses, are reported separately for each outcome in Supplemental Material, Tables B1–B10 (http://dx.doi.org/10.1289/ehp.1003369). Single-city estimates for the percent change in all-cause mortality with a 10-μg/m^3^ increase in BCP and PM_10_ are also presented in Figure 1. Available data were dominated by estimates from the APHEA (Air Pollution and Health–A European Approach) study (Analitis et al. 2006; Atkinson et al. 2001; Katsouyanni et al. 2001; Le Tertre et al. 2002).

**Table 1:** Summary of pooled random effects estimates for PM_10_ and BS from time-series studies.

No. of estimates	Percent change per 10-μg/m^3^ increase (95% CI)			References (Supplemental Material table)*a*
	End point	PM_10_	BS	
Mortality								
All causes*b*		7		0.48 (0.18, 0.79)*		0.68 (0.31, 1.06)*		B, D, E, H (B1)
Cardiovascular		7		0.60 (0.23, 0.97)*		0.90 (0.40, 1.41)*		A, B, H (B2)
Respiratory		7		0.31 (–0.23, 0.86)		0.95 (–0.31, 2.22)		A, B, H (B3)
Hospital admissions								
All respiratory (≥ 65 years)*c*		6		0.70 (0.00, 1.40)*		–0.06 (–0.53, 0.41)		B, C, G (B4)
Asthma + COPD (≥ 65 years)		5		0.86 (0.03, 1.70)*		0.22 (–0.73, 1.18)		C (B5)
Asthma (0–14 years)		5		0.69 (–0.74, 2.14)		1.64 (0.28, 3.02)*		B, C (B6)
Asthma (15–64 years)		5		0.77 (–0.05, 1.61)		0.52 (–0.50, 1.55)		B, C (B7)
Cardiac (all ages)		4		0.51 (0.04, 0.98)*		1.07 (0.27, 1.89)*		B, F (B8)
Cardiac (≥ 65 years)		4		0.67 (0.28, 1.06)*		1.32 (0.28, 2.38)*		F (B9)
IHD (≥ 65 years)		5		0.68 (0.01, 1.36)*		1.13 (0.72, 1.54)*		B, F (B10)
Abbreviations: COPD, chronic obstructive pulmonary disease; IHD, ischemic heart disease. **a**See Supplemental Material, Tables B1–B10 (http://dx.doi.org/10.1289/ehp.1003369), for specific studies. References: A, Analitis et al. (2006); B, Anderson et al. (2001); C, Atkinson et al. (2001); D, Hoek et al. (2000); E, Katsouyanni et al. (2001); F, Le Tertre et al. (2002); G, Prescott et al. (1998); H, Zeghnoun et al. (2001). **b**Includes cardiovascular and respiratory mortality. **c**Includes asthma and COPD. *p < 0.05.								

For most outcomes, pooled effects estimates for a 10-μg/m^3^ increase in exposure are larger for BS than for PM_10_, especially for mortality and hospital admissions for cardiovascular causes (Table 1). However, the average ratio of the IQRs for PM_10_:BS [1.7; see Supplemental Material, Tables B1–B10 (http://dx.doi.org/10.1289/ehp.1003369)] was consistent with the ratios of RR for BS:PM_10_ (e.g., 0.90/0.60 = 1.5 for cardiovascular mortality in Table 1), which suggests that effect estimates expressed for a similar increase in concentration (IQR) would be more or less equivalent. When we used a 10-to-1.1 conversion factor to transform the estimated effect of a 10-μg/m^3^ increase in BS to the estimated effect for a 1-μg/m^3^ increase in EC, the pooled random effect estimate for all-cause mortality changed from 0.68% (95% CI: 0.31, 1.06) to 0.62% (i.e., 0.68/1.1; 95% CI: 0.26, 0.96). When study-specific conversion factors were used, estimated effects for a 1-μg/m^3^ increase in EC ranged from 0.38% to 1.36% (for conversion factors of 1.8 and 0.5, respectively), which suggests that the effect of a 1-μg/m^3^ increase in EC on all-cause mortality is at least eight times larger than the estimated effect of a 1-μg/m^3^ increase in PM_10_ (0.05%).

Studies on BCP and PM_2.5_. Less, but more recent, information was available from studies in which both PM_2.5_ and BCP were measured. Three studies provided estimates of PM_2.5_ and EC, both for all-cause mortality and for cardiovascular mortality. Only two studies provided estimates for respiratory mortality [Cakmak et al. 2009a; Klemm et al. 2004; Mar et al. 2000; Ostro et al. 2007; see Supplemental Material, Tables C1–C3 (http://dx.doi.org/10.1289/ehp.1003369)]. In pooled analyses, a 1-μg/m^3^ increase in PM_2.5_ was associated with a 0.19% increase (95% CI: 0.03, 0.35%) in all-cause mortality and a 0.29% increase (95% CI: 0.07, 0.50%) in cardiovascular mortality. For EC, a 1-μg/m^3^ increase was associated with a 1.45% increase (95% CI: 1.32, 1.57%) in all-cause mortality and 1.77% increase (95% CI: 1.08, 3.08%) in cardiovascular mortality. Thus, expressed per mass unit, effect estimates are much larger (7–8 times) for EC than for PM_2.5_. However, if the ratio of the IQR for PM_2.5_:EC (~ 11) is taken into account, effect estimates were similar.

Available information on the effect of PM and EC on hospital admissions or emergency department visits was even more limited than for mortality, and no pooled estimates could be calculated [Cakmak et al. 2009b; Ostro et al. 2009; Peng et al. 2009; Tolbert et al. 2007; Zanobetti and Schwartz 2006; see Supplemental Material, Table D1 (http://dx.doi.org/10.1289/ehp.1003369)]. When expressed per 1-μg/m^3^ increase, effect estimates were generally 10–30 times higher for EC than for PM_2.5_. However, IQRs for EC were lower by a similar factor. For example, the ratio of the IQRs for PM_2.5_:EC from Zanobetti and Schwartz (2006) (8.9:1.0) was similar to the ratio of the effect estimates for pneumonia with a 1-μg/m^3^ increase in EC:PM_2.5_ (0.054:0.0037), suggesting comparable effects with a comparable change in exposure.

Two-pollutant models of PM mass and BCP. In total, six papers included results of two-pollutant models that included a measure of PM mass as well as BCP. They also included findings on mortality as well as hospital admissions and emergency department visits (Table 2). With one exception, effect estimates for BCP either increased or decreased ≤ 33% after adjusting for PM mass. In contrast, adjusting for BCP substantially reduced most effect estimates for PM mass (effect estimates became negative in three of nine studies and decreased by > 50% in five of the six other studies), suggesting that the effect of BCP is more robust than the effect of PM mass.

**Table 2:** Results from single- and two-pollutant models of time-series studies including PM_10_ or PM_2.5_*a* and BCP (measured as BS in all studies shown here).

Percent change per 10-μg/m^3^ increase (95% CI)						
PM			BS		
Reference (study location)	Health end point	Correlation (*R*) PM–BS*b*	Single-pollutant model	Two-pollutant model*c*	Single-pollutant model	Two-pollutant model*c*
Mortality												
Bremner et al. 1999 (London)		Respiratory mortality		NA		1.3 (0.3, 2.3)		0.4 (–1.0, 1.8)		1.9 (0.2, 3.7)		2.0 (–0.4, 4.4)
		CVD mortality				0.6 (–0.1, 1.2)		0.2 (–0.6, 1.0)		1.2 (0.1, 2.2)		0.8 (–0.6, 2.2)
Hoek et al. 2000 (the Netherlands)		Total mortality		0.77		0.3 (0.0, 0.5)		0.1 (–0.3, 0.6)		0.7 (0.4, 0.9)		0.4 (–0.6, 1.4)
		CVD mortality				0.2 (–0.2, 0.5)		–0.6 (–1.3, 0.1)		0.8 (0.4, 1.2)		2.1 (0.5, 3.7)
Admissions												
Anderson et al. 2001 (West Midlands)		Respiratory admissions (all ages)		0.64		0.6 (–0.5, 1.7)		“Considerably reduced”*d*		1.1 (–0.1, 2.2)		2.0 (0.3, 2.8)
Atkinson et al. 1999a**(London)		A&E visits for asthma; children		NA		2.4 (0.7, 4.1)		2.0 (–0.1, 4.2)		2.8 (–0.0, 5.7)		0.9 (–3.0, 5.1)
Atkinson et al. 1999b (London)*e*		Cardiovascular admissions (> 65 years)		0.6–0.7		0.5 (–0.0, 1.0)		–0.1 (–0.8, 0.5)		1.9 (0.9, 3.0)		2.3 (0.8, 3.8)
Le Tertre et al. 2002 (APHEA)*f*		Cardiac (all ages)		0.5–0.8		0.5 (0.2, 0.8)		–0.2 (–1.2, 0.8)		1.1 (0.4, 1.8)		1.6 (–0.3, 3.5)
		Cardiac (> 65 years)				0.7 (0.4, 1.0)		0.1 (–0.4, 0.7)		1.3 (0.4, 2.2)		1.5 (0.3, 2.7)
		IHD (> 65 years)				0.8 (0.3, 1.2)		0.2 (–0.9, 1.4)		1.1 (0.7, 1.5)		0.8 (–1.1, 2.7)
Abbreviations: A&E, admission and emergency department; CVD, cardiovascular disease; IHD, ischemic heart disease; NA, not available. **a**PM_2.5_ for Anderson et al. (2001); PM_10_ for all other studies. **b**Coefficient of the correlation (*R*) between PM and BS. **c**Two-pollutant models include both PM and BS. **d**Quantitative information not available; paper states that the effect of PM_2.5_ was considerably reduced when BS was included in the model. **e**Results only described qualitatively in the paper; quantitative estimates provided by the authors on request. **f**Study locations were Amsterdam, the Netherlands; Barcelona, Spain; Birmingham, UK; London, UK; and Paris, France.												

Studies on BCP and other PM components. In addition to the effects of BCP compared with PM mass, the relative health effects of BCP compared with other PM components are of interest. Specifically, we were interested in evaluating whether effects of BCP remained significant after the authors had adjusted for other potentially relevant components such as metals. Eight studies that reported effect estimates for EC and PM mass also reported estimates for PM components, including OC, sulfate, and metals (Cakmak et al. 2009a, 2009b; Klemm et al. 2004; Mar et al. 2000; Ostro et al. 2007, 2009; Peng et al. 2009; Sarnat et al. 2008). In general, effects per IQR increase in exposure were greater for EC than for most of the six other frequently reported components [Supplemental Material, Table E1 (http://dx.doi.org/10.1289/ehp.1003369)]. For cardiovascular mortality and morbidity, four of five studies reported significant associations with an IQR increase in OC, four of four reported significant associations with potassium, and three of four reported significant associations with zinc. Estimated effects of an IQR increase in EC on cardiovascular mortality and morbidity were significant in all five studies. For respiratory mortality and morbidity results were more diverse, with the strongest effects observed for EC in two studies (Cakmak et al. 2009a, 2009b) and for OC and sulfate in three studies (Ostro et al 2009; Peng et al. 2009; Sarnat et al. 2008), and no significant (*p* < 0.05) effects for any of the measured components in a sixth study (Ostro et al. 2007).

Three studies also reported estimated effects based on multipollutant models that included a variety of PM components [see Supplemental Material, Table E2 (http://dx.doi.org/10.1289/ehp.1003369)]. Two studies conducted in Santiago, Chile, reported significant associations with mortality (total, cardiac, and respiratory) and hospital admissions (all nonaccidental and respiratory) for EC, OC, and 10–15 of 16 individual elements based on single-pollutant models, but effect estimates for only EC and OC remained significant after adjustment for all other pollutants measured (Cakmak et al. 2009a, 2009b). In a study on emergency department visits for cardiovascular and respiratory disease in 119 U.S. urban communities (Peng et al. 2009), seven major PM components were considered (sulfate, nitrate, silicon, EC, OC, sodium ion, and ammonium ion). These seven components in aggregate constituted 83% of the total PM_2.5_ mass, whereas all other components contributed < 1% individually. In single-pollutant models, cardiovascular admissions were significantly associated with same-day concentrations of five of seven major PM components, including EC. In multipollutant models with all seven components, only EC remained significant. For respiratory admissions, only same-day OC concentrations were significant, both in single-pollutant and in multipollutant models. In a study of associations between hospital admissions for cardiovascular and respiratory diseases in 106 U.S. counties that related admissions to the fraction of 20 elements to the total PM_2.5_ mass rather than the concentration, RRs for cardiovascular and respiratory hospitalizations were highest in counties with high PM_2.5_ content of nickel, vanadium, and EC (Bell et al. 2009). Here, nickel was the most robust component in multipollutant analyses, especially for cardiovascular admissions. Peng et al. (2009) reported statistically significant heterogeneity among effect estimates for different PM components, with the strongest estimated risk of cardiovascular admissions associated with EC concentrations. Cakmak et al. (2009a, 2009b) also reported that the 95% CI of the estimated effect of an IQR increase in EC did not overlap the 95% CIs of the other elements, with the exception of OC and two or three of the other 16 elements, indicating that the effect per IQR for EC was significantly greater than estimated effects of most other single elements.

*Cohort studies on long-term exposure to BCP and PM and mortality and morbidity.* Cohort studies on mortality. We identified seven papers that presented results from four different cohort studies, two of which included effect estimates for BS and PM and two for EC and PM (Table 3). When using the average conversion factor of 10 μg/m^3^ BS = 1.1 μg/m^3^ EC, RRs for all-cause or natural-cause mortality per 1 μg/m^3^ EC in the two European studies and in the study by Smith et al. (2009) ranged from 1.05 to 1.06. RRs for EC and all-cause mortality in the veterans study (Lipfert et al. 2006) were about three times larger than RRs for the same outcomes from the other studies, but because the standard error in the veterans study was two to four times higher compared with the other studies, this study contributes less to the pooled estimate [RR = 1.06 (95% CI: 1.04, 1.09) per μg/m^3^ EC]. Pooled estimates for a 1-μg/m^3^ increase in EC derived using high- and low-end conversion factors of 1.8 and 0.5 μg/m^3^ per 10 μg/m^3^ BS were 1.05 and 1.11, respectively. When expressed per 1 μg/m^3^, the RR for EC is therefore 7–16 times higher than that of PM_2.5_ mass (pooled estimate = 1.007 per 1 μg/m^3^). However, ratios of IQRs for PM_2.5_:EC for the studies by Smith et al. (2009) and Beelen et al. (2008) were 14 and 9, respectively, and we estimated a ratio of about 5 based on graphical data presented for the study by Filleul et al. (2005). For the study by Lipfert et al. (2006), IQRs were not available, but RRs expressed for the difference between the mean concentration and the minimum were 1.06 per 9.5 μg/m^3^ for PM_2.5_ and 1.09 per 0.5 μg/m^3^ for EC. Hence, it appears that effect estimates for PM_2.5_ and EC from cohort studies also would be similar if expressed for an IQR increase in exposure instead of a 1-μg/m^3^ exposure contrast.

**Table 3:** RR for mortality related to long-term exposure to PM_2.5_ and EC per 1 μg/m^3^.

Correlation (*R*) PM–BCP*a*	RR (95% CI)		
	Reference	Cohort	Cause	PM_2.5_	EC
Filleul et al. 2005*b*^,c^		14,284 adults; age 25–59 years; France		0.87*d*		Natural causes*e*		1.010 (1.004, 1.016)		1.06 (1.03, 1.09)
						Cardiopulmonary		1.012 (1.002, 1.023)		1.05 (0.98, 1.11)
						Lung cancer		1.000 (0.983, 1.019)		1.03 (0.93, 1.14)
Lipfert et al. 2006		70,000 male U.S. veterans		0.54		All causes		1.006 (0.993, 1.020)		1.18 (1.05, 1.33)
Beelen et al. 2008*b*		120,852 adults; age 55–69 years; the Netherlands		0.82		Natural causes*e*		1.006 (0.997, 1.015)		1.05 (1.00, 1.10)
						Respiratory		1.007 (0.972, 1.043)		1.20 (0.99, 1.45)
						Cardiovascular		1.004 (0.990, 1.019)		1.04 (0.95, 1.12)
						Lung cancer		1.006 (0.980, 1.033)		1.03 (0.89, 1.18)
						Other		1.008 (0.996, 1.021)		1.04 (0.97, 1.11)
Smith et al. 2009		500,000 adults; age 20–87 years; USA		NA		All causes		1.006 (1.002, 1.010)		1.06 (1.01, 1.11)
						Cardiopulmonary		1.012 (1.008, 1.018)		1.11 (1.03, 1.19)
Pooled effect (random)*f*						All causes		1.007 (1.004, 1.009)		1.06 (1.04, 1.09)
NA, not available. **a**Coefficient of the correlation (*R*) between PM and BCP. **b**RR for EC in European studies estimated from BS as 10 μg/m^3^ BS = 1.1 μg/m^3^. **c**RR for PM_2.5_ estimated from TSP as PM_2.5_ = 0.5 × TSP (Verhoeff et al. 1996; Van der Zee et al. 1998). **d**For all 24 sites, whereas RR presented for 18 sites (nontraffic). **e***International Classification of Diseases, 9th Revision *(World Health Organization 1975), codes < 800. **f**Pooled effect when using 10 μg/m^3^ BS = 1.8 μg/m^3^: 1.05 (95% CI: 1.02, 1.07); when using 10 μg/m^3^ BS = 0.5 μg/m^3^: 1.11 (95% CI: 1.06, 1.16).										

Multipollutant modeling was applied in the studies by Lipfert et al. (2006) and Smith et al. (2009). Based on four-pollutant models that included EC, OC, sulfate, and nitrate, Lipfert et al. (2006) concluded that EC had the greatest estimated impact on all-cause mortality and that nitrate was the next important constituent. In the American Cancer Society (ACS) study, Smith et al. (2009) found that the EC estimate for all-cause mortality was reduced by about 50% and lost statistical significance after adjusting for sulfate and ozone. For cardiopulmonary mortality, EC decreased by about 33% and remained significantly associated after adjustment for sulfate but decreased by about 80% and lost significance after additional adjustment for ozone.

Cohort studies on morbidity. The eight papers on respiratory health outcomes in children included six papers describing results from one Dutch and two German birth cohorts, analyzed using the same exposure assessment strategy, and two papers on lung function growth in two cohorts of Southern California children [Brauer et al. 2002, 2006, 2007; Gauderman et al. 2002, 2004; Gehring et al. 2002; Morgenstern et al. 2007, 2008; see Supplemental Material, Tables F1, F2 (http://dx.doi.org/10.1289/ehp.1003369)]. For most of the studies, PM_2.5_ and BCP were highly correlated (*R* > 0.9). Overall, consistent with other types of studies, estimated effects of a 1-μg/m^3^ increase in BCP were greater than estimated effects of a 1-μg/m^3^ increase in PM_2.5_, whereas effects estimated for IQR increases were similar for BCP and PM_2.5_.

**Figure 2:**
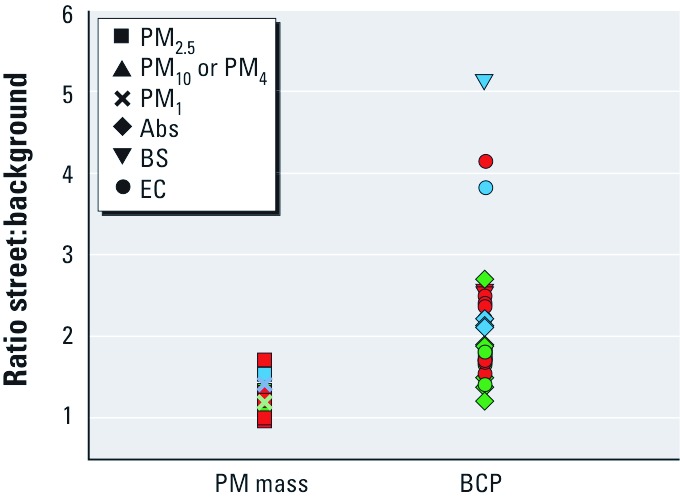
Study-specific street:background ratios for PM mass and BCP concentrations. Blue, ≥ 24-hr average along highways; green, ≥ 24-hr average along inner-city roads; red, daytime and ≤ 12-hr measurements (all inner-city roads). Data from Boogaard et al. (2011), Cyrys et al. (2003), Fischer et al. (2000), Fromme et al. (2005), Funasaka et al. (2000), Harrison et al. (2004), Janssen et al. (1997, 2001, 2008), Kinney et al. (2000), Lena et al. (2002), Riediker et al. (2003), Roemer and van Wijnen (2001b), Roorda-Knape et al. (1998), Roosli et al. (2001), and Smargiassi et al. (2005).

Exposure contrast in BCP compared with PM mass. Street:background ratios were higher and more variable for BCP than for PM mass concentrations [Figure 2; see also Supplemental Material, Table G1 (http://dx.doi.org/10.1289/ehp.1003369)]. On average, BCP concentrations near busy roads were twice as high as urban background BCP concentrations, whereas PM concentrations near busy roads were only about 20% higher than background levels. For all single sites, the street:background ratio for BCP was higher than the corresponding ratio for PM mass. For the studies included in Figure 2, the average roadside increment of EC relative to PM_2.5_ was 55% (95% CI: 46%, 63%) when the conversion 10 μg/m^3^ BS = 1.1 μg/m^3^ EC was used. Using the lower and upper conversion factors of 0.5 and 1.8 resulted in an average percentage of 41% (95% CI: 29%, 54%) and 70% (95% CI: 59%, 82%), respectively [see Supplemental Material, Table G2 (http://dx.doi.org/10.1289/ehp.1003369)].

Comparison of calculated health benefits of traffic abatement measures using PM_2.5_ or BCP. The estimated percentage EC in the roadside increment in PM_2.5_ of 40–70% implies that every 1-μg/m^3^ reduction in traffic-related PM_2.5_ along busy roads will result in a 0.4- to 0.7-μg/m^3^ reduction in EC. When the average conversion factor of 10 μg/m^3^ BS = 1.1 μg/m^3^ EC is used to derive the RR for a 1-μg/m^3^ increase in EC and the percentage of EC in a roadside increment of PM_2.5_, the increase in life expectancy per person is five times higher for EC than for PM_2.5_ (3.6 months vs. 21 days; Table 4). When the maximum and minimum conversion factors of 1.8 and 0.5 μg/m^3^ EC per 10 μg/m^3^ BS are used, the increase in estimated life expectancy is four to nine times higher. Therefore, estimated health benefits are much larger when expressed in terms of EC compared with an equivalent change in PM mass.

**Table 4:** Comparison of the estimated effect on life expectancy of reductions in PM_2.5_ mass and EC resulting from a traffic management plan.

Component	Conversion of BS to EC*a*	RR*b*	Reduction [μg/m^3^ (95% CI)]*c*	Increase in life expectancy per person*d*
PM_2.5_				1.007		1.00		21 days
EC		10 μg/m^3^ BS = 1.1 μg/m^3^ EC		1.06		0.55 (0.46, 0.63)		3.6 months (3.0, 4.1 months)
		10 μg/m^3^ BS = 1.8 μg/m^3^ EC		1.05		0.70 (0.59, 0.82)		3.1 months (2.6, 3.6 months)
		10 μg/m^3^ BS = 0.5 μg/m^3^ EC		1.11		0.41 (0.29, 0.54)		4.5 months (3.2, 5.9 months)
**a**BS was converted to EC for two of the four studies that were used to calculate the RR for EC and for 8 of 16 studies that were used to calculate the percentage EC in the roadside increment of PM_2.5_ over background. **b**RR per 1-μg/m^3^. **c**A traffic abatement measure is evaluated that reduces EC proportional to the percentage EC in the roadside increment of PM_2.5_ over background [Supplemental Material, Table G2 (http://dx.doi.org/10.1289/ehp.1003369)]. **d**Values in parentheses are based on the 95% CI for the reduction.								

## Discussion

Our review shows that health effect estimates from mortality and morbidity time-series studies as well as cohort studies were higher for BCP than for PM_10_ or PM_2.5_ when expressed for a 1-μg/m^3^ increase in exposure and similar when expressed for an IQR increase in exposure. A relatively large part (40–70%) of the roadside increment in PM_2.5_ mass concentrations can be attributed to BCP. Based on the calculated RRs for all-cause mortality from cohort studies as well as the estimated percentage EC in the roadside increment in PM_2.5_ mass, the estimated increase in life expectancy associated with a hypothetical traffic abatement policy measure was four to nine times higher than when expressed per achievable reduction in BCP compared with the estimated effect of an equivalent reduction in PM_2.5_ mass.

*Health effects of BCP compared with PM mass.* Single-pollutant effect estimates for daily mortality or hospital admissions generally were an order of magnitude higher for BCP than for PM_10_ and PM_2.5_ when expressed per 1 μg/m^3^. When differences in IQRs were accounted for, effect estimates were generally similar. It should be noted that there was a moderate to moderately high correlation between PM_10_ and BS measurements reported by the individual studies included in the pooled estimates (Pearson correlations of 0.5–0.8), consistent with correlations between daily wintertime PM_10_ and BS concentrations from a study in 14 European study areas (Hoek et al. 1997). Although this raises concerns about the ability to distinguish effects due to PM_10_ versus BS, there is at least some variation in the temporal patterns of these exposures.

In studies examining a variety of different PM components, BCP generally showed significant associations, especially with cardiovascular health end points, both before and after adjusting for other components. For cohort studies, pooled estimates for all-cause mortality per 1 μg/m^3^ were 5–14 times higher for BCP than for PM_2.5_, but IQRs for PM _2.5_ were higher than those for BCP by a similar factor.

The implication of similar effects per IQR is that for policies that reduce all relevant components of PM proportional to current levels, estimated health benefits would be similar based on either indicator. The IQR-based comparison is also the relevant comparison for health impacts assessments of general air quality. However, for assessments of exposure conditions dominated by combustion sources, or policies directed toward specific combustion sources, the comparison of RRs expressed per unit change in mass is relevant.

The available evidence from two-pollutant models for time-series studies suggests that the effect of BCP is more robust than the effect of PM mass. However, two-pollutant models with BCP and PM mass were not conducted in any of the cohort studies. Although overall the results of multipollutant analysis including BCP, sulfate, and ozone in the ACS study suggest that sulfate has the most robust association with all-cause and cardiopulmonary mortality, Smith et al. (2009) indicate that this can also be caused by differential amounts of measurement error. In the ACS study, where exposure was assessed at the metropolitan area level, estimates of the spatial distribution of EC likely have more measurement error than the assigned sulfate exposures because EC is more locally generated than is sulfate, which is a secondary pollutant with little spatial variation. When measurement error is present, variables measured with high precision will tend to dominate model-based predictions relative to variables measured with less precision (Smith et al. 2009). For time-series studies, there are no large differences in temporal relationships between central-site ambient concentrations and personal exposure for BCP and PM_2.5_ (Janssen et al. 2005). In addition, issues related to the correlation between different pollutants and the extent to which they can act as surrogates for the etiologic agent(s) complicate the interpretation of results from multipollutant models (Tolbert et al. 2007). Our interpretation that the results from two-pollutant models for the time-series studies suggest that BCP is a more health–relevant indicator in these studies than is PM mass is also supported by Roemer and van Wijnen (2001a, 2002), who calculated separate effect estimates with separate exposure estimates using background and traffic-influenced measurement stations and for the total population and people living along busy roads. Effect estimates for urban background BS were larger in the population living along busy roads than for the total population, suggesting that this subpopulation was more highly exposed. Indeed, effect estimates for the population living along busy roads using BS measured at traffic stations were more or less equivalent to effect estimates for the total population using BS measured at urban background stations.

Further evidence of health effects of primary combustion is obtained in studies that use source apportionment techniques to assess associations of particles from different sources with health. Particles from traffic or local combustion were associated with daily mortality and hospital admissions (Cakmak et al. 2009a, 2009b; Laden et al. 2000; Mar et al. 2000; Sarnat et al. 2008). Although measures of BCP are not frequently used in human controlled exposure studies, several human exposure studies using exposure to diesel exhaust have documented airway and systemic inflammation (Salvi et al. 2000), as well as responses that provide a possible mechanism for cardiac events such as myocardial infarction (Mills et al. 2007). Two studies illustrated the importance of particle composition: Mills et al. (2008) found little effect of 2-hr exposure to high PM_2.5_ concentrations taken in Edinburgh, attributed to the high sea salt content (90%); Urch et al. (2005) found blood pressure increases in healthy subjects related to the OC content of fine PM—largely from motorized traffic—but not to total PM_2.5_.

*Spatial contrast in BCP compared with PM_2.5_.* Higher street:background ratios for BCP compared with PM (Figure 2) are consistent with the larger impact of traffic on BCP than on PM mass concentrations. However, the studies included in our review represent a variety of settings, including different distances to the roadside, traffic densities (including vehicle types), averaging times, seasons, and meteorological conditions. These factors probably (partly) explain the variability in ratios observed between studies.

The impact of traffic on BCP was also demonstrated for temporal concentration variations by Schaap and van der Gon (2007), who showed that BS levels on rural and urban locations in the Netherlands were about 50% higher on weekdays than on Sundays, whereas BS concentrations at urban traffic locations were about 100% higher on weekdays than on Sundays. Comparison of weekdays and Sundays for PM_10_ mass concentrations showed much smaller differences (5–15%). We estimated that, on average, 55% of the roadside increment in PM_2.5_ consisted of EC based on absolute differences in concentrations between street and background concentrations. Deriving an overall quantitative estimate of this percentage across studies is complicated by the different measurement methods used for BCP, in which differences between methods for measuring EC and differences in conversion of optical measures of BCP to EC concentrations both need to be taken into account. We therefore converted BS and Abs to EC using a conversion factor based on the average of 11 identified area-specific comparison studies and used the range of these 11 values in sensitivity analyses.

Our estimates compare well with previous studies (Lefebvre et al. 2011; Lena et al. 2002; Millstein and Harley 2010). In a study on the spatial variation in EC and PM_2.5_ in relation to local truck-traffic density, Lena et al. (2002) estimated that EC represents 52% of the total PM_2.5_ generated by large trucks. In comparison, in a modeling study on the effects of retrofitting trucks with diesel particle filters, EC accounted for 64% of total diesel PM_2.5_ emissions (Millstein and Harley 2010). Similarly, in a modeling study of the effect of a speed limit reduction on traffic-related EC, Lefebvre et al. (2011) estimated that EC traffic emissions account for 70% of the total PM_2.5_ exhaust emissions. These figures are also in the range provided by the European emission inventory guidebook for the EC fraction in PM_2.5_ in exhaust emissions for different vehicle categories (e.g., passenger cars, vans, and trucks) (Ntziachristos and Samaras 2009). The contribution of BCP in roadside increments of PM_10_ will be smaller because resuspended road dust, including brake and tire wear, results in a more substantial contribution to PM_10_ (Gietl et al. 2010; Janssen et al. 1997).

*Comparison of calculated health benefits of traffic abatement measures using PM_2.5_ or BCP.* We evaluated the gain in life expectancy of a 1-μg/m^3^ decrease in PM_2.5_ and 0.55 μg/m^3^ EC, based upon the average contribution of EC to the increment in PM concentration in studies comparing a major road and urban background. This calculation can be interpreted as an indication of the potential difference in a health impact assessment based upon PM_2.5_ or BCP for populations living along a major road. It can also be interpreted as the potential health gain for policies that reduce concentrations in approximately the same ratio as the current roadside increment, for example, a limitation of overall traffic intensity.

There are few empirical data to support larger impacts of policies on BCP than on PM_2.5_ mass. In an evaluation of the effects of retrofitting trucks with diesel engine particle filters on air quality in Southern California, Millstein and Harley (2010), using an Eulerian photochemical air quality model, estimated a decrease in EC concentrations in 2014 of 12–14%. The estimated effect on PM_2.5_ mass concentrations was much smaller (< 1%). In a modeling study of the effect of a speed limit reduction (from 120 to 90 km/hr) on air quality in Flanders, EC concentrations decreased up to 30% just next to the busiest highways, compared with an estimated reduction of at most 8.5% for PM_2.5_. For buffer zones of 0–100 m distance to the highways EC concentrations decreased by 9–10% (Lefebvre et al. 2011). A small monitoring study on the effects of road closures associated with the 2004 Democratic National Convention in Boston suggested slightly lower concentrations of EC and NO_2_ during the road closure periods at monitoring sites proximate to the closed highway segments. This decrease was not observed for PM_2.5_ or farther from major highways (Levy et al. 2006).

Our finding of a larger increase in life expectancy associated with a hypothetical traffic abatement policy measure when expressed per achievable reduction in BCP than when expressed per an equivalent amount of PM_2.5_ mass illustrates that health effects of such policies may be seriously underestimated when based upon PM_2.5_ or PM_10_. As an illustration, we calculated the increase in life expectancy for the population living along major roads. We did not attempt to calculate the impact for the larger population. However, although the absolute improvement of air quality will be smaller, we expect that the differences between BCP and PM mass will be similar. In a modeling study of the effect of a speed limit reduction, Lefebvre et al. (2011) estimated a decrease in EC concentrations of 0.4 μg/m^3^ for buffer zones within 200 m of the highways, affecting about 75,000 inhabitants if the abatement measure would be implemented on all highways in Flanders and Brussels. For the buffer zone within 1,500 m of the highway the reduction was smaller, 0.17 μg/m^3^ (5%), but affecting up to 1.8 million inhabitants.

*Overall discussion.* Our review shows that BC is associated with health effects that are not reflected quantitatively in the same way by particle mass, as indicated by the higher effect estimates per 1 μg/m^3^ for BCP compared with PM mass.

In the reviewed studies, ambient measurements of various BCP metrics were used. Although motorized traffic was an important source of BC in most of these studies, they included the impact of all combustion sources on BCP concentrations, including coal and wood burning, shipping emissions, and industrial sources. In a review of source apportionment studies for fine PM EC, Schauer (2003) found that the combined contribution of diesel- and gasoline-powered vehicles ranged from 74% to 98%; the contribution from biomass burning ranged from 0.7% to 25%; and the contribution from other sources, from 0.5% to 17%. The derived risks therefore represent those for BCP as a general indicator of combustion particles, not exclusively traffic. Issues therefore remain when these risk estimates are applied to specific combustion sources such as traffic or wood burning. We however hold that BCP more closely resembles the harmful components in these air pollution mixtures than does general PM_2.5_.

Associations between individual elements and mortality or morbidity could be explained by the health effects of that element or the health effects of a pollution mixture of which the element is an indicator. Therefore, BCP could be serving as an indicator for the larger category of primary combustion particles, which, in addition to BCP, can include trace metals and hydrocarbons such as polycyclic aromatic hydrocarbons, any or all of which could be acting to cause adverse health effects. Our analysis assumes that these other components are equally decreased relative to reductions in BCP when measures are taken that reduce emissions of combustion particles. This assumption will be more valid for measures that do not affect engine characteristics, such as a restriction of the number of vehicles, compared with measures that affect particle composition, such as speed reduction or changes in engine types or fuel mixtures. Furthermore, because BCP is a marker for tailpipe emissions, it is less suitable to evaluate the health benefits of traffic-oriented abatement measures that are expected to result in reductions in nontailpipe emissions, from brake linings, crank cases, tire wear, and so forth, which may be uncorrelated with reductions in BCP.

In 2003, a WHO working group recommended reevaluation of BS as part of the reconsideration of the WHO air quality guidelines and consideration of the addition of photometric analysis of BCP on the PM_2.5_ filters (WHO 2003). Our review supports this recommendation. We foresee application of a BCP indicator in evaluation of current levels of traffic-related air pollution, wood smoke, or other combustion particles and policies aimed at reducing these sources. Of the different methods to measure BCP, probably the best method would be EC measurements using standard methodology (Cavalli et al. 2010). However, it is beyond the scope of this article to make recommendations about the methods that should be used to measure BCP.

In summary, we do not promote BCP as an alternative marker for PM mass because this would disregard the effects of coarse particles or particles from other sources. Nonetheless, our review shows that BCP is a valuable additional air quality indicator that would be particularly useful to evaluate health risks of air pollution dominated by primary combustion emissions, as well as benefits of traffic abatement measures.

## Supplemental Material

(201 KB) PDF
