# Supplementary material for: Black Carbon as an Additional Indicator of the Adverse Health Effects of Airborne Particles Compared with PM10 and PM2.5
Source: Environ Health Perspect. 2011 Aug 2;119(12):1691–9. doi: 10.1289/ehp.1003369 (PMC3261976; doi:10.1289/ehp.1003369)
Supplement: (201 KB) PDF [file ehp.1003369.s001.pdf]

Supplemental Material

Black Carbon as an Additional Indicator of the Adverse Health Effects of Airborne Particles Compared to PM_{10} and $PM_{2.5}$

Nicole AH Janssen, Gerard Hoek, Milena Simic-Lawson, Paul Fischer, Leendert van Bree, Harry ten Brink, Menno Keuken, Richard W Atkinson, H Ross Anderson, Bert Brunekreef, Flemming R Cassee

Tables of contents

Table A1. Estimation of elemental carbon (EC) from black smoke or absorbance of $PM_{2.5}$ filters ... 3
Single city estimates for mortality and hospital admissions in studies that include both PM_{10} and Black Smoke ... 4
Table B1. All cause mortality; all age ... 4
Table B2. CVD mortality; all age ... 5
Table B3. Respiratory mortality; all age ... 6
Table B4. Respiratory hospital admissions, age ≥65 ... 7
Table B5. Respiratory hospital admissions; Asthma and COPD, age ≥65 ... 7
Table B6. Respiratory hospital admissions, Asthma, age 0-14 ... 8
Table B7. Respiratory hospital admissions: Asthma, age 15-64 ... 8
Table B8. Hospital admissions: Cardiac, age ≥ 65 ... 9
Table B9. Hospital admissions: Cardiac, age ≥ 65 ... 9
Table B10. Hospital admission; IHD, age ≥ 65 ... 10
Table C1. Effect estimates for $PM_{2.5}$ and EC for all cause mortality ... 11
Table C2. Effect estimates for PM2.5 and EC for cardiovascular mortality ... 11
Table C3. Effect estimates for $PM_{2.5}$ and EC on respiratory mortality ... 11
Table D1. Effect estimates for $PM_{2.5}$, EC and sulfate on hospital admissions and emergency department visits ... 12
Table E1. Effect estimates for EC and other particle components ... 13
Table E2. Results from single and multi-pollutant models including BCP and sulfate ... 14
Effects of $PM_{2.5}$ and BCP in cohort studies of respiratory health in children ... 15
Table F1: Effects of $PM_{2.5}$ and BCP in birth cohort studies ... 15
Table F2: Effects of $PM_{2.5}$ and BCP in cohort studies on lung function growth ... 17
Table G1. Contrasts between traffic and background locations for BCP and $PM_{2.5}$ ... 18
Table G2. Estimated percentage EC in the roadside increment of $PM_{2.5}$ ... 20
References ... 21

Table A1. Estimation of elemental carbon (EC) from black smoke or absorbance of $PM_{2.5}$ filters

Reference	Study period	locations	Measurement method EC	BCP	R[a]	Regression equation Intercept	slope	Increase in EC per 10 µg/m³ increase in BS[b]
Edwards et al. 1983	NA	Washington, US; urban + traffic	Thermal optical [c]	BS	0.82	-0.1	0.13	1.3
Erdman et al. 1993	1989/ 1990	Berlin; Germany; urban	VDI 3481	BS	0.93	0	0.23	1.8
Schaap et al. 2007	1998/99; 2001/02	Netherlands, urban	Sunset	BS	0.92	0.32	0.09	0.9
		Netherlands, rural	Sunset	BS	0.87	0.16	0.06	0.6
Kinney et al. 2000	1996	New York, US urban + traffic	Sunset	Abs	0.95	0	0.83	0.8
Janssen et al. 2001[d]	1997/ 1998	Netherlands; urban+traffic	VDI 2465-part 1	Abs	0.92	0	2.11	1.7
Lena et al. 2002	1999	New York, US urban + traffic	Sunset	Abs	0.90	0	0.52	0.5
Adams et al. 2002	1999/ 2000	London, UK; urban + traffic	NIOSH	Abs	0.98	0	1.21	1.2
Cyrys et al. 2003	1999/ 2000	Munich, Germany urban+traffic	VDI 2465-part 1	Abs	0.97	-1.19	2.02	1.6
Cyrys et al. 2003	1999/ 2000	Netherlands, rural, urban, traffic	VDI 2465-part 1	Abs	0.97	-0.26	1.61	1.3
Cyrys et al. 2003	1999/ 2000	Sweden, rural, urban, traffic	VDI 2465-part 1	Abs	0.85	0.36	0.90	0.7
							**Mean[e]**	**1.1**
							**Min**	**0.5**
							**Max**	**1.8**

[a] coefficient of the correlation between EC and BCP concentrations

[b] : Results from studies that have used the VDI protocol were divided by 1.25, as this method has been shown to overestimate EC by on average 25% (Schmid et al. 2001); An increase in 1 unit of Abs is considered to equal an increase of 10 µg/m³ BS, according to Roorda-Knape et al. 1998

[c] measurement method not further specified

[d] paper presents regression equation as Abs = EC; inverse equation, forced through zero, calculated using the original data from the study

[e] Mean, minimum and maximum of all 11 values; mean for BS only = 1.16 (range: 0.6-1.8; n=4); mean for Abs only = 1.12 (range0.5-1.7; n=7)

Supplement B:

Single city estimates for mortality and hospital admissions in studies that include both PM_{10} and Black Smoke

Table B1. All cause mortality; all age

(cities in italics occur more than once; city in bold included in meta-analysis)

		Estimate PM_{10}		Estimate BS		IQR ($\mu g/m^3$)		Concentration[a]		Corr (R)	Period	Selected
**Reference**	**City**	**Beta**	**SE**	**Beta**	**SE**	**PM_{10}**	**BS**	**PM_{10}**	**BS**	**PM-BS[b]**		**lag[c]**
Verhoeff, 1996	*Amsterdam*	0.00060	0.00038	0.00171	0.00077	22	10	38	12	0.51	1986-92	Lag0
Roemer, 2001	*Amsterdam*	0.00027	0.00020	0.00324	0.00093	18	7	39	10	NA	1987-94	Lag1
Katsouyanni, 2001	Athens[e]	0.00153	0.00028	0.00065	0.00012	*NA*	*NA*	40	64	NA	1992-96	Lag0-1
Katsouyanni, 2001	**Barcelona**	0.00093	0.00018	0.00157	0.00027	24 [d]	18 [d]	60	39	NA	1991-96	Lag0-1
Katsouyanni, 2001	Birmingham	0.00028	0.00026	0.00034	0.00047	15 [d]	7 [d]	21	11	NA	1992-96	Lag0-1
Katsouyanni, 2001	Cracow[e]	0.00013	0.00035	-0.00021	0.00021	*NA*	*NA*	54	36	NA	1990-96	Lag0-1
Zeghnoun, 2001a	**Le Havre**	0.00079	0.00057	0.00026	0.00085	24	12	36	16	0.70	1990-95	PM lag1 / BS lag 0-1
Katsouyanni, 2001	***London***	0.00069	0.00017	0.00093	0.00030	14[d]	8 [d]	25	11	NA	1992-96	Lag0-1
Bremner, 1999	*London*	0.00026	0.00023	0.00074	0.00038	*NA*	*NA*	28	13	NA	1992-94	Lag1
Hoek, 2000	**Netherlands**	0.00018	0.00008	0.00040	0.00010	23 [d]	9 [d]	34	10	0.77	1986-94	Lag1
Zeghnoun, 2001b	*Paris*	0.00066	0.00020	0.00043	0.00015	15	14	22	16	NA	1990-95	Lag1
Katsouyanni, 2001	***Paris***	0.00043	0.00023	0.00038	0.00015	13 [d]	15 [d]	22	21	NA	1991-96	Lag0-1
Zeghnoun, 2001a	**Rouen**	0.00024	0.00040	0.00035	0.00083	21	14	33	19	0.73	1990-95	Lag1
Anderson, 2001	**West Midlands**	0.00008	0.00042	0.00036	0.00064	*NA*	*NA*	23	13	0.64	1994-96	Lag0-1
**% change per 10 $\mu g/m^3$ increase**		**%**	**95% CI**	**%**	**95% CI**							
Pooled Fixed Effects		0.34	(0.23-0.47)	0.52	(0.37-0.66)							
Pooled Random Effects		0.48	(0.18-0.79)	0.68	(0.31-1.06)							
Heterogeneity chi-squared (df=6)		Q=19.9	p=0.003	Q=19.2	P=0.004							

[a] Mean or median ($\mu g/m^3$)
[b] Coefficient of the correlation between PM_{10} and BS concentrations
[c] In case multiple lags were reported in the paper, we used the estimate discussed by the author, as indicated in APED as 'selected lag'
[d] Taken from APHEA II paper on hospital admissions (le Tetre, 2002)
[e] Excluded from meta-analyses because PM_{10} was partly derived from BS

Table B2. CVD mortality; all age

(cities in italics occur more than once; city in bold included in meta-analysis)

		Estimate PM_{10}		Estimate BS		IQR (μg/m^3)		Concentration[a]		Corr (R)	Period	Selected
**Reference**	**City**	**Beta**	**SE**	**Beta**	**SE**	**PM_{10}**	**BS**	**PM_{10}**	**BS**	**PM-BS[b]**		**lag[c]**
Analitis, 2006	Athens[e]	0.00167	0.00045	0.00069	0.00018	*NA*	*NA*	40	64	NA	1992-96	Lag0-1
Analitis, 2006	**Barcelona**	0.00055	0.00032	0.00137	0.00050	24 [b]	18 [d]	60	39	NA	1991-96	Lag0-1
Analitis, 2006	Birmingham	0.00021	0.00040	0.00039	0.00071	15 [b]	7 [d]	21	11	NA	1992-96	Lag0-1
Analitis, 2006	Cracow[e]	0.00032	0.00052	-0.00007	0.00031	*NA*	*NA*	54	36	NA	1990-96	Lag0-1
Zeghnoun, 2001a	**Le Havre**	0.00252	0.00126	0.00164	0.00155	24	12	36	16	0.70	1990-95	PM lag1; BS lag0-3
Analitis, 2006	***London***	0.00091	0.00028	0.00156	0.00046	14 [d]	8 [d]	25	11	NA	1992-96	Lag0-1
Bremner, 1999	*London*	0.00055	0.00031	0.00117	0.00066	*NA*	*NA*	28	13	NA	1992-94	Lag1
Hoek, 2000	*Netherlands*	0.00019	0.00018	0.00079	0.00020	23 [d]	9 [d]	34	10	0.77	1986-94	lag 0-6
Analitis, 2006	***Netherlands***	0.00017	0.00016	0.00026	0.00027	23 [d]	9 [d]	33	9	NA	1990-95	Lag0-1
Hoek, 2001	*Netherlands*	0.00015	0.00018	0.00071	0.00020	23 [d]	9 [d]	34	10	0.77	1992/86-1994[f]	lag 0-6
Zeghnoun, 2001b	*Paris*	0.00086	0.00037	0.00036	0.00029	15	14	22	16	NA	1990-95	PM lag 2; BS lag 1
Analitis, 2006	***Paris***	0.00081	0.00047	0.00063	0.00029	13 [d]	15 [d]	22	21	NA	1991-96	Lag0-1
Zeghnoun, 2001a	**Rouen**	0.00106	0.00069	0.00276	0.00155	21	14	33	19	0.73	1990-95	Lag1
Anderson, 2001	**West Midlands**	0.00041	0.00061	0.00089	0.00092	*NA*	*NA*	23	13	0.64	1994-96	Lag0-1
**% change per 10 μg/m^3 increase**		**%**	**95% CI**	**%**	**95% CI**							
Pooled Fixed Effects		0.45	(0.22-0.68)	0.73	(0.41-1.06)							
Pooled Random Effects		0.60	(0.23-0.97)	0.90	(0.40-1.41)							
Heterogeneity chi-squared (df=6)		Q=9.9	p=0.127	Q=10.2	p=0.116							

[a] Mean or median (μg/m^3)
[b] Coefficient of the correlation between PM_{10} and BS concentrations
[c] In case multiple lags were reported in the paper, we used the estimate discussed by the author, as indicated in APED as 'selected lag'
[d] Taken from APHEA II paper on hospital admissions (le Tetre, 2002)
[e] Excluded from meta-analyses because PM_{10} was partly derived from BS
[f] 1992-1994 for PM_{10} ; 1986-1994 for BS

Table B3. Respiratory mortality; all age

(cities in italics occur more than once; city in bold included in meta-analysis)

Reference	City	Estimate PM_{10} Beta	Estimate PM_{10} SE	Estimate BS Beta	Estimate BS SE	IQR ($\mu g/m^3$) PM_{10}	IQR ($\mu g/m^3$) BS	Concentration[a] PM_{10}	Concentration[a] BS	Corr (R) PM-BS[b]	Period	Selected lag[c]
Analitis, 2006	Athens[e]	0.00101	0.00122	0.00006	0.00048	*NA*	*NA*	40	64	NA	1992-96	Lag0-1
Analitis, 2006	**Barcelona**	0.00117	0.00075	0.00374	0.00100	24 [d]	18 [d]	60	39	NA	1991-96	Lag0-1
Analitis, 2006	Birmingham	0.00003	0.00078	0.00069	0.00130	15 [d]	7 [d]	21	11	NA	1992-96	Lag0-1
Analitis, 2006	Cracow[e]	0.00529	0.00216	0.00357	0.00132	*NA*	*NA*	54	36	NA	1990-96	Lag0-1
Zeghnoun, 2001a	**Le Havre**	0.00200	0.00196	0.00249	0.00294	24	12	36	16	0.70	1990-95	PM lag2; BS lag0-1
Analitis, 2006	***London***	0.00022	0.00044	-0.00034	0.00073	14 [d]	8 [d]	25	11	NA	1992-96	Lag0-1
Bremner, 1999	*London*	0.00128	0.00050	0.00190	0.00084	*NA*	*NA*	28	13	NA	1992-94	Lag3
Analitis, 2006	***Netherlands***	0.00031	0.00036	0.00029	0.00061	23 [d]	9 [d]	33	9	NA	1990-95	Lag0-1
Dab, 1996	*Paris*	0.00155	0.00059	0.00069	0.00048	NA	NA	51	32	NA	1987-92	PM lag0-1; BS lag1
Analitis, 2006	***Paris***	-0.00121	0.00095	0.00063	0.00029	13 [d]	15 [d]	22	21	NA	1991-96	Lag0-1
Zeghnoun, 2001a	**Rouen**	0.00176	0.00120	0.00201	0.00310	21	14	33	19	0.73	1990-95	Lag0-1
Anderson, 2001	**West Midlands**	-0.00058	0.00100	0.00006	0.00153	*NA*	*NA*	23	13	0.64	1994-96	Lag0-1

**% change per 10 $\mu g/m^3$ increase**	**%**	**95% CI**	**%**	**95% CI**
Pooled Fixed Effects	0.31	(-0.16-0.78)	0.70	(-0.05-1.45)
Pooled Random Effects	0.31	(-0.23-0.86)	0.95	(-0.31-2.22)
Heterogeneity chi-squared (df=6)	Q=6.9	p=0.329	Q=12.5	p=0.051

[a] Mean or median ($\mu g/m^3$)

[b] Coefficient of the correlation between PM_{10} and BS concentrations

[c] In case multiple lags were reported in the paper, we used the estimate discussed by the author, as indicated in APED as 'selected lag'

[d] Taken from APHEA II paper on hospital admissions (le Tetre, 2002)

[e] Excluded from meta-analyses because PM_{10} was partly derived from BS

Table B4. Respiratory hospital admissions, age ≥65

(cities in italics occur more than once; city in bold included in meta-analysis)

Reference	City	Estimate PM_{10} Beta	Estimate PM_{10} SE	Estimate BS Beta	Estimate BS SE	IQR (µg/m³) PM_{10}	IQR (µg/m³) BS	Concentration[a] PM_{10}	Concentration[a] BS	Corr (R) PM-BS[b]	Period	Selected lag[c]
Atkinson, 2001	**Barcelona**	0.00198	0.00060	-0.00070	0.00083	24	18	56	39	0.5-0.8[d]	1994-96	Lag0-1
Atkinson, 2001	Birmingham	0.00090	0.00061	0.00286	0.00115	15	7	25	13	0.5-0.8[d]	1992-94	Lag0-1
Prescott, 1998	**Edinburgh**	0.00208	0.00304	0.00305	0.00338	NA	NA	21	9	0.4	1992-95	Lag1-3
Atkinson, 2001	***London***	0.00040	0.00036	-0.00111	0.00068	14	8	28	13	0.5-0.8[d]	1992-94	Lag0-1
Atkinson, 1999	*London*	0.00096	0.00041	0.00082	0.00063	NA	NA	29	13	0.6-0.7	1992-94	Lag3
Atkinson, 2001	**Netherlands**	0.00119	0.00025	0.00000	0.00036	23	9	40	13	0.5-0.8[d]	1992/89-1995[e]	Lag0-1
Atkinson, 2001	**Paris**	-0.00010	0.00062	0.00050	0.00046	13	15	23	23	0.5-0.8[d]	1992-96	Lag0-1
Anderson, 2001	**West midlands**	-0.00045	0.00069	-0.00018	0.00100	*NA*	*NA*	23	13	0.64	1994-96	Lag0-1
**% change per 10 µg/m³ increase**												
Pooled Fixed Effects		0.85	(0.49-1.20)	-0.07	(-0.58-0.44)							
Pooled Random Effects		0.70	(0.00-1.40)	-0.06	(-0.53-0.41)							
Heterogeneity chi-squared (df=5)		Q=13.1	P=0.023	Q=5.4	p=0.372							

Table B5. Respiratory hospital admissions; Asthma and COPD, age ≥65

(cities in italics occur more than once; city in bold included in meta-analysis)

Reference	City	Estimate PM_{10} Beta	Estimate PM_{10} SE	Estimate BS Beta	Estimate BS SE	IQR (µg/m³) PM_{10}	IQR (µg/m³) BS	Concentration[a] PM_{10}	Concentration[a] BS	Corr (R) PM-BS[b]	Period	Selected lag[c]
Atkinson, 2001	**Barcelona**	0.00257	0.00080	-0.00212	0.00116	24	18	56	39	0.5-0.8[d]	1994-96	Lag0-1
Atkinson, 2001	**Birmingham**	0.00050	0.00097	0.00218	0.00199	15	7	25	13	0.5-0.8[d]	1992-94	Lag0-1
Atkinson, 2001	***London***	0.00030	0.00056	0.00040	0.00103	14	8	28	13	0.5-0.8[d]	1992-94	Lag0-1
Atkinson, 1999	*London*	0.00227	0.00137	-0.00091	0.00099	NA	NA	29	13	0.6-0.7	1992-94	Lag 3
Atkinson, 2001	**Netherlands**	0.00109	0.00030	0.00070	0.00046	23	9	40	13	0.5-0.8[d]	1992/89-1995[e]	Lag0-1
Atkinson, 2001	**Paris**	-0.00060	0.00098	0.00020	0.00077	13	15	23	23	0.5-0.8[d]	1992-96	Lag0-1
**% change per 10 µg/m³ increase**												
Pooled Fixed Effects		0.95	(0.48-1.42)	0.36	(-0.81-1.54)							
Pooled Random Effects		0.86	(0.03-1.70)	0.22	(-0.73-1.18)							
Heterogeneity chi-squared (df=4)		Q=8.3	p=0.08	Q=6.0	p=0.199							

[a] Mean or median (µg/m³); [b] Coefficient of the correlation between PM_{10} and BS concentrations;
[c] In case multiple lags were reported in the paper, we used the estimate discussed by the author, as indicated in APED as 'selected lag'
[d] Range in correlation coefficient for all 8 cities described in Atkinson et al (2001) (3 cities not included in this review as no data on black smoke was available)
[e] 1992-1995 for PM_{10}; 1989-1995 for BS

Table B6. Respiratory hospital admissions, Asthma, age 0-14

(cities in italics occur more than once; city in bold included in meta-analysis)

Reference	City	Estimate PM_{10} Beta	Estimate PM_{10} SE	Estimate BS Beta	Estimate BS SE	IQR ($\mu g/m^3$) PM_{10}	IQR ($\mu g/m^3$) BS	Concentration[a] PM_{10}	Concentration[a] BS	Corr (R) PM-BS	Period	Selected lag
Atkinson, 2001	**Barcelona**	0.00266	0.00392	0.00989	0.00484	24	18	56	39	0.5-0.8[c]	1994-96	Lag0-1
Atkinson, 2001	Birmingham	0.00276	0.00100	0.00198	0.00199	15	7	25	13	0.5-0.8[c]	1992-94	Lag0-1
Atkinson, 2001	***London***	0.00060	0.00072	0.00109	0.00123	14	8	28	13	0.5-0.8[c]	1992-94	Lag0-1
Atkinson, 1999	*London*	0.00324	0.00203	0.00245	0.00179	NA	NA	29	13	0.6-0.7	1992-94	Lag3
Atkinson, 2001	**Netherlands**	-0.00090	0.00062	0.00139	0.00091	23	9	40	13	0.5-0.8[c]	1992/89-1995[e]	Lag0-1
Atkinson, 2001	**Paris**	0.00070	0.00113	0.00090	0.00087	13	15	23	23	0.5-0.8[c]	1992-96	Lag0-1
Anderson, 2001	**West midlands**	0.00797	0.00321	0.00714	0.00329	*NA*	*NA*	23	13	0.64	1994-96	Lag0-1
**% change per 10 $\mu g/m^3$ increase**												
Pooled Fixed Effects		0.24	(-0.56-1.05)	1.47	(0.41-2.54)							
Pooled Random Effects		0.69	(-0.74-2.14)	1.64	(0.28-3.02)							
Heterogeneity chi-squared (df=4)		Q=9.5	P=0.050	Q=5.6	p=0.231							

Table B7. Respiratory hospital admissions: Asthma, age 15-64

(cities in italics occur more than once; city in bold included in meta-analysis)

Reference	City	Estimate PM_{10} Beta	Estimate PM_{10} SE	Estimate BS Beta	Estimate BS SE	IQR ($\mu g/m^3$) PM_{10}	IQR ($\mu g/m^3$) BS	Concentration[a] PM_{10}	Concentration[a] BS	Corr (R) PM-BS[b]	Period	Selected lag[c]
Atkinson, 2001	**Barcelona**	0.00040	0.00202	0.00208	0.00121	24	18	56	39	0.5-0.8[d]	1994-96	Lag0-1
Atkinson, 2001	Birmingham	0.00247	0.00121	0.00276	0.00239	15	7	25	13	0.5-0.8[d]	1992-94	Lag0-1
Atkinson, 2001	***London***	0.00139	0.00076	0.00178	0.00137	14	8	28	13	0.5-0.8[d]	1992-94	Lag0-1
Atkinson, 1999	*London*	0.00555	0.00249	0.00234	0.00224	NA	NA	29	13	0.6-0.7	1992-94	PM Lag3; BS lag2
Atkinson, 2001	**Netherlands**	0.00040	0.00066	-0.00040	0.00093	23	9	40	13	0.5-0.8[d]	1992/89-1995[e]	Lag0-1
Atkinson, 2001	**Paris**	0.00119	0.00097	0.00080	0.00076	13	15	23	23	0.5-0.8[d]	1992-96	Lag0-1
Anderson, 2001	**West midlands**	-0.00233	0.00419	-0.00284	0.00432	*NA*	*NA*	23	13	0.64	1994-96	Lag0-1
**% change per 10 $\mu g/m^3$ increase**												
Pooled Fixed Effects		0.77	(-0.05-1.61)	0.52	(-0.50-1.55)							
Pooled Random Effects		0.77	(-0.05-1.61)	0.52	(-0.50-1.55)							
Heterogeneity chi-squared (df=4)		Q=2.2	P=0.697	Q=3.1	p=0.549							

[a] Mean or median ($\mu g/m^3$); [b] Coefficient of the correlation between PM_{10} and BS concentrations

[c] In case multiple lags were reported in the paper, we used the estimate discussed by the author, as indicated in APED as 'selected lag'

[d] Range in correlation coefficient for all 8 cities described in Atkinson et al (2001) (3 cities not included in this review as no data on black smoke was available)

[e] 1992-1995 for PM_{10}; 1989-1995 for BS

Table B8. Hospital admissions: Cardiac, age ≥ 65

Reference	City	Estimate PM_{10} Beta	Estimate PM_{10} SE	Estimate BS Beta	Estimate BS SE	IQR (μg/m³) PM_{10}	IQR (μg/m³) BS	Concentration[a] PM_{10}	Concentration[a] BS	Corr (R) PM-BS[b]	Period	Selected lag[c]
Le Tertre, 2002	**Barcelona**	0.00050	0.00046	0.00066	0.00064	24	18	56	39	0.5-0.8[d]	1994-96	Lag0-1
Le Tertre, 2002	Birmingham	-0.00014	0.00039	0.00114	0.00078	15	7	25	13	0.5-0.8[d]	1992-94	Lag0-1
Le Tertre, 2002	**London**	0.00104	0.00027	0.00214	0.00049	14	8	28	13	0.5-0.8[d]	1992-94	Lag0-1
Le Tertre, 2002	**Paris**	0.00020	0.00028	0.00057	0.00022	13	15	23	23	0.5-0.8[d]	1992-96	Lag0-1
Anderson, 2001	**West midlands**	0.00030	0.00108	0.00169	0.00117	*NA*	*NA*	23	13	0.64	1994-96	Lag0-1
**% change per 10 μg/m³ increase**												
Pooled Fixed Effects		0.54	(0.21-0.87)	0.83	(0.47-1.19)							
Pooled Random Effects		0.51	(0.04-0.98)	1.07	(0.27-1.89)							
Heterogeneity chi-squared (df=3)		Q=5.7	p=0.129	Q=8.8	p=0.032							

[a] Mean or median (μg/m³); [b] Coefficient of the correlation between PM_{10} and BS concentrations
[c] In case multiple lags were reported in the paper, we used the estimate discussed by the author, as indicated in APED as 'selected lag'
[d] Range in correlation coefficient for all 8 cities described in Le Tertre et al (2002) (no information on cardiac admissions available for the Netherlands; 3 other cities not included in this review as no data on black smoke was available)

Table B9. Hospital admissions: Cardiac, age ≥ 65

Reference	City	Estimate PM_{10} Beta	Estimate PM_{10} SE	Estimate BS Beta	Estimate BS SE	IQR (μg/m³) PM_{10}	IQR (μg/m³) BS	Concentration[a] PM_{10}	Concentration[a] BS	Corr (R) PM-BS[b]	Period	Selected lag[c]
Le Tertre, 2002	**Barcelona**	0,00068	0.00055	0.00130	0.00075	24	18	56	39	0.5-0.8[d]	1994-96	Lag0-1
Le Tertre, 2002	**Birmingham**	0,00031	0.00047	0.00168	0.00094	15	7	25	13	0.5-0.8[d]	1992-94	Lag0-1
Le Tertre, 2002	**London**	0,00096	0.00032	0.00227	0.00057	14	8	28	13	0.5-0.8[d]	1992-94	Lag0-1
Le Tertre, 2002	**Paris**	0,00053	0.00035	0.00042	0.00027	13	15	23	23	0.5-0.8[d]	1992-96	Lag0-1
**% change per 10 μg/m³ increase**												
Pooled Fixed Effects		0.67	(0.28-1.06)	0.86	(0.41-1.30)							
Pooled Random Effects		0.67	(0.28-1.06)	1.32	(0.28-2.38)							
Heterogeneity chi-squared (df=3)		Q=1.5	p=0.673	Q=9.9	p=0.019							

[a] Mean or median (μg/m³); [b] Coefficient of the correlation between PM_{10} and BS concentrations
[c] In case multiple lags were reported in the paper, we used the estimate discussed by the author, as indicated in APED as 'selected lag'
[d] Range in correlation coefficient for all 8 cities described in Le Tertre et al (2002) (no information on cardiac admissions available for the Netherlands; 3 other cities not included in this review as no data on black smoke was available)

Table B10. Hospital admission; IHD, age ≥ 65

(cities in italics occur more than once; city in bold included in meta-analysis)

Reference	City	Estimate PM_{10} Beta	Estimate PM_{10} SE	Estimate BS Beta	Estimate BS SE	IQR (μg/m³) PM_{10}	IQR (μg/m³) BS	Concentration[a] PM_{10}	Concentration[a] BS	Corr (R) PM-BS[b]	Period	Selected lag[c]
Le Tertre, 2002	**Barcelona**	-0.00087	0.00087	0.00061	0.00120	24	18	56	39	0.5-0.8[d]	1994-96	Lag0-1
Le Tertre, 2002	Birmingham	0.00033	0.00076	-0.00073	0.00150	15	7	25	13	0.5-0.8[d]	1992-94	Lag0-1
Le Tertre, 2002	***London***	0.00104	0.00049	0.00265	0.00086	14	8	28	13	0.5-0.8[d]	1992-94	Lag0-1
Atkinson, 1999	*London*	0.00298	0.00128	0.00288	0.00119	NA	NA	29	13	0.6-0.7	1992-94	PM lag0; BS Lag3
Le Tertre, 2002	**Netherlands**	0.00036	0.00018	0.00100	0.00026	23	9	40	13	0.5-0.8[d]	1992/89-1995[b]	Lag0-1
Le Tertre, 2002	**Paris**	0.00168	0.00057	0.00116	0.00043	13	15	23	23	0.5-0.8[d]	1992-96	Lag0-1
Anderson, 2001	**West Midlands**	0.00208	0.00209	0.00198	0.00220	*NA*	*NA*	23	13	0.64	1994-96	Lag0-1
**% change per 10 μg/m³ increase**												
Pooled Fixed Effects		0.50	(0.20-0.81)	1.13	(0.72-1.54)							
Pooled Random Effects		0.68	(0.01-1.36)	1.13	(0.72-1.54)							
Heterogeneity chi-squared (df=4)		Q=8.8	p=0.066	Q=3.6	p=0.463							

[a] Mean or median (μg/m³)
[b] Coefficient of the correlation between PM_{10} and BS concentrations
[c] In case multiple lags were reported in the paper, we used the estimate discussed by the author, as indicated in APED as 'selected lag'
[d] Range in correlation coefficient for all 8 cities described in Le Tertre et al (2002) (no information on cardiac admissions available for the Netherlands; 3 other cities not included in this review as no data on black smoke was available)

Supplement C: Study specific effect estimates for mortality in studies that include both $PM_{2.5}$ and EC

Table C1. Effect estimates for $PM_{2.5}$ and EC for all cause mortality

Reference	City	Estimate $PM_{2.5}$ Beta	Estimate $PM_{2.5}$ SE	Estimate EC Beta	Estimate EC SE	IQR $PM_{2.5}$	IQR EC	Concentration[a] $PM_{2.5}$	Concentration[a] EC	Corr (R) PM-EC[b]	Period	Selected lag[c]
Klemm, 2004[b]	Atlanta	0.00544	0.00184	0.01343	0.01072	11.6	1.1	19.6	2.0	NA	1998-2000	Lag01
Ostro, 2007[b]	6 California counties	0.00056	0.00037	0.00829	0.00776	14.6	0.8	19.3	1.0	0.53	2000-2003	Lag3
Cakmak, 2009	Santiago, Chile	0.00212	0.00025	0.01440	0.00063	35.8	5.3	NA	3.3	NA	1998-2006	PM NA; EC lag1
**% change per 1 µg/m^3 increase[c]**												
Pooled Fixed Effects		0.17	(0.13-0.21)	1.45	(1.32-1.57)							
Pooled Random Effects		0.19	(0.03-0.35)	1.45	(1.32-1.57)							

[a] Mean or median; [b] Coefficient of the correlation between $PM_{2.5}$ and EC concentrations;
[c] In case multiple lags were reported in the paper, we used the estimate discussed by the author, as indicated in APED as 'selected lag'; [d] age >65 ; not all age;
[d] please note that in supplement B the % change was calculated per 10 µg/m^3

Table C2. Effect estimates for PM2.5 and EC for cardiovascular mortality

Reference	City	Estimate PM2.5 Beta	Estimate PM2.5 SE	Estimate EC Beta	Estimate EC SE	IQR $PM_{2.5}$	IQR EC	Concentration[a] $PM_{2.5}$	Concentration[a] EC	Corr (R) PM-EC[b]	Period	Selected lag[c]
Mar, 2000	Phoenix	0.00685	0.00236	0.04400	0.01820	8.5	1.2	12.0	1.3	0.84	1995-1997	Lag1
Ostro, 2007[b]	6 California counties	0.00105	0.00054	0.02574	0.01129	14.6	0.8	19.3	1.0	0.53	2000-2003	Lag3
Cakmak, 2009	Santiago, Chile	0.00327	0.00037	0.01736	0.00097	35.8	5.3	NA	3.3	NA	1998-2006	PM NA; EC lag1
**% change per 1 µg/m^3 increase[c]**												
Pooled Fixed Effects		0.26	(0.20-0.32)	1.76	(1.57-1.96)							
Pooled Random Effects		0.29	(0.07-0.50)	1.77	(1.08-3.08)							

[a] Mean or median; [b] Coefficient of the correlation between $PM_{2.5}$ and EC concentrations;
[c] In case multiple lags were reported in the paper, we used the estimate discussed by the author, as indicated in APED as 'selected lag'; [d] age >65 ; not all age;
[d] please note that in supplement B the % change was calculated per 10 µg/m^3

Table C3. Effect estimates for $PM_{2.5}$ and EC on respiratory mortality

Reference	City	Estimate $PM_{2.5}$ Beta	Estimate $PM_{2.5}$ SE	Estimate EC Beta	Estimate EC SE	IQR $PM_{2.5}$	IQR EC	Concentration[a] $PM_{2.5}$	Concentration[a] EC	Corr (R) PM-EC[b]	Period	Selected lag[c]
Ostro, 2007[b]	6 California counties	0.00098	0.00103	-0.03298	0.02219	14.6	0.8	19.3	1.0	0.53	2000-2003	Lag3
Cakmak, 2009	Santiago, Chile	0.00648	0.00058	0.03453	0.00146	35.8	5.3	NA	3.3	NA	1998-2006	PM NA; EC lag2

[a] Mean or median; [b] Coefficient of the correlation between $PM_{2.5}$ and EC concentrations;
[c] In case multiple lags were reported in the paper, we used the estimate discussed by the author, as indicated in APED as 'selected lag'; [d] age >65 ; not all age;
[d] please note that in supplement B the % change was calculated per 10 µg/m^3

Supplement D: Study specific effect estimates for hospital admissions and emergency department visits in studies that include both $PM_{2.5}$ and EC

Table D1. **Effect estimates for $PM_{2.5}$, EC and sulfate on hospital admissions and emergency department visits.**
(significant effects ($p<0,05$) in bold)

Reference	City	Endpoint	$PM_{2.5}$		EC		Sulfate		IQR (µg/m^3)			Concentration (µg/m^3)		
			beta	se	beta	se	beta	se	$PM_{2.5}$	EC	Sulfate	$PM_{2.5}$	EC	Sulfate
Hospital admissions														
Zanobetti, 2006	Boston; elderly	Pneumonia	**0.0037**	0.0015	**0.0540**	0.0159			8.9	1.0		11.1	1.2	
		AMI	**0.0048**	0.0021	**0.0391**	0.0194								
Ostro, 2009	6 california counties; children	All respiratory	**0.0027**	0.0008	**0.0640**	0.0277	**0.0199**	0.0089	14.6	0.8	1.5	19.4	1.0	2.0
		Asthma	0.0023	0.0015	0.0628	0.0339	0.0026	0.0199						
		Bronchitis	**0.0034**	0.0015	0.0525	0.0341	**0.0449**	0.0211						
		Pneumonia	0.0022	0.0014	0.0623	0.0476	0.0182	0.0224						
Peng, 2009	119 US Counties; elderly	CVD	**0.00068**	0.00021	**0.01794**	0.00375	**0.00140**	0.00075	9.5	0.4	3.1	12.2	0.6	2.6
		Respiratory	0.00031	0.00035	0.00998	0.00599	0.00266	0.00140						
Emergency department visits														
Tolbert, 2007	Atlanta; All age[a]	CVD	0.00046	0.00056	**0.01295**	0.00439	-0.00026	0.00161	11.0	1.2	3.8	17.1	1.6	4.9
		Respiratory	0.00046	0.00047	-0.00349	0.00313	0.00183	0.00147						
Cakmak, 2009[b]	Santiago, Chile; All age	All non-accid.	**0.00152**	0.00018	**0.02287**	0.00184	**0.02232**	0.00804	40.3	4.8	2.3	NA	2.8	2.6
		Respiratory	**0.00241**	0.00028	**0.03531**	0.00247	**0.03185**	0.01092						

[a] also estimates from additional endpoints available from 3 older papers that included a shorter study period (Metzger et al. 2004; Sarnat et al. 2008; Tolbert et al. 2000)
[b] sulfate estimated from S

Table E1. Effect estimates for EC and other particle components

Effects expressed as % increase per IQR, (significant effects (p<0,05) in bold)

Reference	City	Endpoint	% increase per IQR							IQR (µg/m³)						
			EC	OC	Sulfate	Nitrate	Zn	K	Si	EC	OC	Sulfate	Nitrate	Zn	K	Si
Mortality																
Mar, 2000[a]	Phoenix	All cause	ns	ns	**-3.0**		ns	ns		1.2	3.0	0.8		na	0.06	
		Cardiovascular	**5.2**	**4.4**	ns		ns	**3.2**								
Klemm, 2004	Atlanta	All cause	1.5	1.3	3.4	-0.1				1.1	2.4	3.9	1.3			
Ostro, 2007	6 California counties	All cause	0.7	0.6	0.2	0.1	0.6	0.2	0.0	0.8	4.6	1.5	5.5	0.01	0.08	0.15
		Cardiovascular	**2.1**	1.6	0.6	1.5	**2.2**	**0.5**	0.6							
		Respiratory	-2.6	-2.9	1.1	1.0	-0.5	0.5	1.5							
Maynard, 2007	Boston	All cause	**2.3**		**1.1**					0.2		2.3				
		Respiratory	**3.7**		2.1											
		Cardiovascular	1.5		-0.2											
		Stroke	4.4		2.0											
		Diabetes	5.7		2.9											
Cakmak, 2009[a]	Santiago, Chile	All cause	**7.9**	**6.6**	**3.2**		**5.3**	**3.5**	1.7	5.3	7.4	2.8		0.08	0.23	0.20
		Cardiac	**9.6**	**8.3**	**5.1**		**5.9**	**5.1**	**4.2**							
		Respiratory	**20.0**	**17.9**	**6.9**		**13.6**	**11.7**	**8.1**							
Hospital admission																
Ostro, 2009	6 California counties; children	All respiratory	**5.4**	**3.4**	**3.0**	**3.3**	1.6	0.8	**2.8**							
		Asthma	5.3	4.0	0.4	2.4	1.8	0.3	2.9	0.8	4.5	1.5	5.6	0.01	0.08	0.15
		Bronchitis	4.4	**4.8**	**6.9**	3.9	1.7	2.1	**6.1**							
		Pneumonia	5.3	**4.5**	2.8	2.2	**2.0**	0.7	4.3							
Peng, 2009	119 US counties; elderly	Cardiovascular	**0.7**	**0.7**	0.4	**0.5**			**0.2**	0.4	3.2	3.1	1.6			0.07
		Respiratory	0.4	**0.8**	-0.3	0.0			0.1							
Emergency department visits																
Sarnat, 2008[b]	Atlanta; all age	CVD	**2.5**	**2.4**	0.7	0.2	**1.3**	**3.0**	0.8				Not available			
		Respiratory	-0.4	-0.3	**2.0**	-0.1	-0.3	0.2	-0.4							
Cakmak, 2009[c]	Santiago, Chile; all age	All non-accid.	**11.5**	**9.3**	**5.2**		**5.2**	**5.8**	**5.8**	4.8	8.5	2.3		0.07	0.21	0.18
		Respiratory	**18.3**	**14.3**	**7.5**		**7.5**	**9.8**	**11.4**							

[a] ns = non-significant (effect estimates not reported in paper)

[b] estimates from Sarnat (2008) used instead of Tolbert (2007), despite shorter period (4 instead of 6 years) as the Sarnat paper included more other elements.

[c] sulfate estimated from S ;

Table E2. Results from single and multi-pollutant models including BCP and sulfate

Ref. / city	Health endpoint	BCP metric	R Sulfate-BCP[a]	% change in RR[b]			
				Sulfate single	Sulfate multi	BCP single	BCP multi
Hoek, 2000	Total mortality	BS	0.65	3.2 (0.6 to 5.9)	2.7 (-0.3 to 5.8)	2.8 (1.7 to 3.8)	1.2 (-1.5 to 4.1)
	CVD mortality			2.1 (-1.9 to 6.3)	0.8 (-3.7 to 5.4)	3.2 (1.6 to 4.8)	2.9 (-1.3 to 7.4)
Anderson, 2001; West Midlands	Respiratory admissions	BS	0.30	0.8 (-1.3 to 2.9)	Na	2.1 (-0.1 to 4.2)	2.4 (0.1 to 4.7)
Maynard, 2007	Total mortality	BC	0.44	1.1 (0.01 to 2.0)	0.5 (-0.45 to 1.6)	2.3 (1.2 to 3.4)	2.2 (0.2 to 4.2)
Peng, 2009	Respiratory admissions	EC	0.18	-0.3 (-1.1 to 0.5)	-0.6 (-1.1 to 0.3)	0.4 (-0.1 to 0.9)	0.0 (-0.1 to 0.8)
119 US Counties[c]	Cardiovascular admissions			0.4 (-0.0 to 0.9)	0.0 (-0.5 to 0.6)	0.7 (0.4 to 1.0)	0.8 (0.3 to 1.3)
Cakmak, 2009a;	Total mortality	EC	0.33	3.2 (1.4 to 5.0)	Lost significance	7.9 (7.2 to 8.6)	Remained
Santiago, Chile[d]	Cardiac mortality			5.1 (2.4 to 8.0)		9.6 (8.5 to 10.8)	significantly
	Respiratory mortality			6.9 (1.9 to 12.1)		20.0 (18.2 to 21.9)	associated
Cakmak, 2009b;	All non-accidental adm.	EC	0.20	5.2 (1.5 to 9.1)	Lost significance	11.5 (9.6 to 13.5)	Remained
Santiago, Chile[c]	Respiratory admissions			7.5 (2.4 to 12.8)		18.3 (15.6 to 21.2)	significant

[a] Coefficient of the correlation between sulfate and BCP concentrations;
[b] RRs expressed as reported in the paper: IQR for Maynard (2007); Peng (2009) and Cakmak (2009a; 2009b); 1 to 9th percentile for Hoek (2000); 10-90th percentile for Anderson (2001);
[c] Multi-pollutant estimates also adjusted for OCM, Nitrate, Silicon, Sodium_ion and Ammonium
[d] Multi-pollutant estimates also adjusted for 16 other PM components and 3 gases; quantitative estimates for multi-pollutant models requested from the authors, but not received

Supplement F:

Effects of PM$_{2.5}$ and BCP in cohort studies of respiratory health in children

Table F1: Effects of PM$_{2.5}$ and BCP in birth cohort studies

Reference	Cohort	R PM-BCP[a]	RR expressed per	Health endpoint[b]	RR PM		RR BCP	
Gehring, 2002	Birth cohort (GINI / LISA) 1756 children born in Munich city Age 2	0.96	Expressed per IQR: PM$_{2.5}$: 1.5 µg/m^3 Abs: 0.4 m^{-1} x10^{-5}	Wheeze	0.96	(0,83-1,12)	0.98	(0.84-1.14)
				Dry cough at night	**1,20**	(1.02-1.42)	1.16	(0.98-1.37)
				DD obstr/spast/ astmoid bronchitis	0.92	(0.78-1.09)	0.94	(0.79-1.12)
				Respiratory infections	0.98	(0.80-1.20)	0.99	(0.80-1.22)
				Sneezing/runny stuffed nose	0.96	(0.82-1.12)	0.92	(0.78-1.09)
Brauer, 2002	Piama cohort; 3000 children throughout the Netherlands; symptoms at age 2	0.99	Expressed per IQR: PM$_{2.5}$: 3.2 µg/m^3 Abs: 0.54 m^{-1} x10^{-5}	Wheeze	1.14	(0.98-1.34)	1.11	(0.97-1.26)
				DD-asthma	1.12	(0.84-1.50)	1.12	(0.88-1.43)
				Dry cough at night	1.04	(0.88-1.23)	1.02	(0.88-1.17)
				DD bronchitis	1.04	(0.85-1.26)	0.99	(0.84-1.17)
				E,N,T infections	**1.20**	(1.01-1.42)	**1.15**	(1.00-1.33)
				DD flu/serious colds	**1.12**	(1.00-1.27)	1.09	(0.98-1.21)
				Itchy rash	1.01	(0.88-1.16)	1.02	(0.91-1.15)
				DD eczema	0.95	(0.83-1.10)	0.96	(0.85-1.08)
Brauer, 2006	Birth cohort (Piama); 3000 children through-out the Netherlands	0.99	Expressed per IQR: PM$_{2.5}$: 3 µg/m^3; EC: 0.5 µg/m^3	Otitis media				
				Age 1	1.13	(0.98- 1.32)	1.11	(0.98- 1.26)
				Age 2	**1.13**	(1.00-1.27)	**1.10**	(1.00-1.22
	Birth cohort (LISA), 600 children from Munich, Germany		Expressed per IQR: PM$_{2.5}$: 3 µg/m^3; EC: 0.5 µg/m^3	Otitis media				
				Age 1	1.19	(0.73- 1.92)	1.12	(0.83- 1.51)
				Age 2	1.24	(0.84-1.83)	1.10	(0.86-1.41)
Brauer, 2007	PIAMA cohort; 3000 children throughout the Netherlands; symptoms at age 4	0.99	Expressed per IQR: PM$_{2.5}$: 3.3 µg/m^3 Abs: 0.58 m^{-1} x10^{-5}	Wheeze	1.20	(0.99-1.46)	**1.18**	(1.00-1.40)
				DD-asthma	1.32	(0.98-1.71)	1.30	(0.98-1.71)
				Dry cough at night	1.14	(0.98-1.33)	**1.14**	(1.00-1.31)
				DD bronchitis	0.86	(0.66-1.11)	0.88	(0.69-1.11)
				E,N,T infections	**1.17**	(1.02-1.34)	**1.16**	(1.03-1.31)
				DD flu/serious colds	**1.25**	(1.07-1.46)	**1.19**	(1.04-1.37)
				Itchy rash	0.98	(0.85-1.14)	0.97	(0.85-1.10)
				DD eczema	0.98	(0.82-1.17)	0.97	(0.83-1.14)

Morgenstern, 2007[c]	GINI / LISA cohort 3577 children residing in the Munich metropolitan area; age 2	0.49	Expressed per IQR: $PM_{2.5}$: 1.0 µg/m^3 Abs: 0.22 m^{-1} x10^{-5}	Wheeze	1.10	(0.96-1.25)	1.09	(0.90-1.33)
				Dry cough at night	1.03	(0.89-1.19)	1.18	(0.93-1.50)
				DD obstr/spast/ astmoid bronchitis	1.05	(0.92-1.20)	0.85	(0.31-2.34)
				Respiratory infections	1.09	(0.94-1.27)	1.05	(0.79-1.39)
				Sneezing/runny stuffed nose	**1.19**	(1.04-1.36)	**1.27**	(1.04-1.56)
Morgenstern, 2008[c]	GINI / LISA cohort ±3000 children residing in the Munich metropolitan area; age 4 and 6	0.49	Expressed per IQR: $PM_{2.5}$: 1.0 µg/m^3 Abs: 0.22 m^{-1} x10^{-5}	DD obstr/spast/ astmoid bronchitis	1.12	(0.94-1.29)	**1.56**	(1.03-2.37)
				DD hay fever	1.01	(0.91-1.12)	**1.59**	(1.11-2.27)
				DD eczema	1.00	(0.86-1.24)	1.03	(0.86-1.24)
				PR obstr/spast/ astmoid bronchitis	0.97	(0.91-1.02)	0.96	(0.83-1.11)
				DD hay fever	1.02	(0.96-1.08)	1.11	(0.93-1.31)
				DD eczema	1.05	(0.90-1.37)	1.05	(0.93-1.47)
				Prevalent asthma	**1.26**	(1.04-1.51)	**1.20**	(1.02-1.42)
				Incident asthma	**1.28**	(1.10-1.49)	**1.21**	(1.06-1.38)
				Asthma symptoms	**1.15**	(1.02-1.28)	**1.12**	(1.01-1.24)
				Wheeze	**1.20**	(1.08-1.33)	**1.16**	(1.06-1.27)
				Sneezing, runny/blocked nose	**1.12**	(1.01-1.24)	**1.11**	(1.01-1.21)
				Hayfever	1.05	(0.83-1.32)	1.04	(0.85-1.27)
				Atopic eczema	1.00	(0.90-1.11)	1.00	(0.91-1.10)
				At age 8 years				
				Wheeze	**1.29**	(1.04-1.62)	**1.22**	(1.00-1.48)
				BHR	0.98	(0.76-1.24)	1.04	(0.84-1.29)
				Allergic sensitization	1.16	(0.96-1.39)	1.12	(0.95-1.32)
				- In utero exposure	**1.02**	(1.00-1.03)	**1.08**	(1.02-1.15)
				- First-year exposure	1.01	(0.99-1.03)	**1.14**	(1.01-1.29)

[a] Coefficient of the correlation between $PM_{2.5}$ and BCP concentrations;
[b] DD = doctor diagnosed; PR = parental report
[c] further analyses of Gehring et al. (2002). Here, the study population was expanded by also including subjects who lived outside the Munich area. Although this resulted in a lower correlation between $PM_{2.5}$ and BCP (R=0.49), the performance of the land use regression model used to assign exposure to individual participants was poorer than that of the smaller population (Morgenstern et al. 2007).

Table F2: Effects of $PM_{2.5}$ and BCP in cohort studies on lung function growth

**Reference**	**Cohort**	**R PM-BCP**[a]	**RR expressed per**	**Health endpoint**	**RR PM**		**RR BCP**	
Gauderman, 2002	Results from 2 cohorts 1) 1457 children Recruited 1993 4 year follow-up	0,91	Expressed for concentration range (max - min) $PM_{2.5}$: 22.2 µg/m^3 EC: 1.1 µg/m^3	Growth rate FVC (%)	-0.42	(-0.86- 0.03)	**-0.49**	(-0.88- -0.09)
				Growth rate FEV1 (%)	-0.63	(-1.28- 0.02)	**-0.71**	(-1.30- -0.12)
				Growth rate MMEF (%)	-0.94	(-1.88- 0.01)	**-1.07**	(-1.94- -0.19)
	2) 1678 children Recruited 1996 4 year follow-up	0,93		Growth rate FVC (%)	-0.14	(-0.67- 0.40)	-0.17	(-0.67- 0.33)
				Growth rate FEV1 (%)	-0.39	(-1.06- 0.28)	-0.40	(-1.02- 0.23)
				Growth rate MMEF (%)	**-0.94**	(-1.87- 0.00)	**-0.92**	(-1.78- -0.05)
Gauderman, 2004	Cohort 1) 8 years follow-up	0,91	Expressed for concentration range (max - min) $PM_{2.5}$: 22.8 µg/m^3 EC: 1.1 µg/m^3	Growth rate FVC (ml)	-60.1	(-166.1- 45.9)	-77.7	(-166.7- 11.3)
				Growth rate FEV1 (ml)	**-79.7**	(-153.0- -6.4)	**-87.9**	(-146.4- -29.4)
				Growth rate MMEF (ml)	-168.9	(-345.5- 7.8)	**-165.5**	(-323.4- -7.6)

[a] Coefficient of the correlation between $PM_{2.5}$ and BCP concentrations;

Table G1. Contrasts between traffic and background locations for BCP and $PM_{2.5}$

- Ratios and differences values in bold were provided in the paper; values in regular print were calculated from the paper; grey for footnote A

Reference	Location / period	Site characteristics: Traffic site	Site characteristics: Background sites	Measurement method: PM	Measurement method: BCP	Averaging time / # observations	Mean concentration at traffic site[a]: PM	Mean concentration at traffic site[a]: BCP	Mean concentration at background site[a]: PM	Mean concentration at background site[a]: BCP	Ratio traffic/ background: PM	Ratio traffic/ background: BCP
Janssen, 1997	Arnhem, The Netherlands / Oct–Nov 1994	Curbside (0,5 m); 15,000 veh/day;	Urban; 200 m from nearest busy road	$PM_{2.5}$ mass	Black smoke	8 h (8:30 – 16:30)<br>28 paired observations	42.9	51.0	35.0	22.7	**1.3**	**2.6**
Roorda-Knape, 1998	Delft (1) and Rotterdam (2), Netherlands / May-aug 1995	1) 15 m from highway; 131.907 veh/day	305 m from the same highway	$PM_{2.5}$ mass	Black smoke	1 week; 10 paired observations	20.1	14.9	18.5	7.4	1.09	2.01
		2) 32 m from highway; 132.559 veh/day	260 m from the same highway			1 week; 8 paired observations	20.8	12.2	19.6	8.7	1.07	1.40
Roemer, 2001	Amsterdam, Netherlands / Jan 1998 – March 1999	12-14m from highway (94.000 veh/day)	Urban background site	PM_1 TEOM	Black smoke	24h; 65 days with complete information on all 3 sites	14	36	10	7	1.4	5.14
		7 m from busy street (30.000 veh/day)					12	18	10	7	1.2	2.57
Fischer, 2000	Amsterdam, Netherlands / Jan-Apr 1995	Outside 18 homes in main streets (5.951-30.974 veh/day)	Outside 18 homes in side street (<3.000 veh/day)	$PM_{2.5}$ mass	Abs. of $PM_{2.5}$ filters	24h; 1-2 samples per home; 18 days with ≥ 1 obs at both types of homes	25.0	2.8	21.0	1.5	**1.20**	**1.84**
Janssen, 2001; 2008[b]	Outside 24 schools<400m of highways in NL. (1) Apr 1997 - May '98; (2) Nov 2001-Oct '02	50 m from busy highway ; ±140.000 veh/day; 10% trucks	301 (period 1) or 375 (period 2) m distance of highway with low traffic (50.000 veh/day)	$PM_{2.5}$	Abs. of $PM_{2.5}$ filters;	Annual average, calculated from 5-10 week measurements per site (adjusted for temporal variation at reference site)	19.6 (1)<br>17.5 (2)	1.94 (1)<br>2.31 (2)	17.5 (1)<br>15.1 (2)	1.00 (1)<br>1.38 (2)	1.12 (1)<br>1.16 (2)	2.23 (1)<br>1.81 (2)
		300 m from highway ; ±110.000 veh/day; 17% trucks					21.8 (1)<br>17.4 (2)	1.51(1)<br>1.97 (2)	17.5 (1)<br>15.1 (2)	1.00 (1)<br>1.38 (2)	1.25 (1)<br>1.15 92)	1.66 (1)<br>1.51 (2)
		200 m from highway with; ±100.000 veh/day; 10% trucks					17.5 (1)<br>16.2 (2)	1.21(1)<br>1.91 (2)	17.5 (1)<br>15.1 (2)	1.00 (1)<br>1.38 (2)	1.00 (1)<br>1.07 (2)	1.27 (1)<br>1.46 (2)
Lena, 2002	New York, USA July – Aug 1999	Intersection along truck route (515 veh/h; 24% large trucks)	Garden of home in residential street; no-truck traffic zone	$PM_{2.5}$	EC estimated from Abs of $PM_{2.5}$ filters (using 12 co-located EC measurements)	10-12h; starting at 6:00 each day; 2-6 samples per traffic site; 9 days at control site (corresponding values at control site calculated from table)	29.9	5.86	17.7	2.34	1.69	2.50
		Intersection along truck route; highly congested (783 veh/h; 35% large trucks)					28.2	7.34	21.6	3.04	1.31	2.41
		Intersection along truck route, spacious and open (657 veh/h; 23% large trucks)					20.7	3.8	20.8	2.2	1.00	1.73
Smargiassi 2005	Montreal, Canada / May – June, YEAR: NA	<10 m of a major urban residential arteries (20.457 veh/day)	Quiet residential street	$PM_{2.5}$	Abs of $PM_{2.5}$ filter	24h; 7 weeks; weekdays only; all sites simultaneously	13.7	1.42	12.4	1.18	1.11	1.20
		<10 m of a major urban residential arterie (32.713 veh/day					13.4	1.63	12.4	1.18	1.08	1.38
		On a collector artery; 19.137 veh/day on collector; >150.000 on highway					15.4	2.50	12.4	1.18	1.24	2.12

Reference	Location / period	Site characteristics		Measurement method		Averaging time / # observations	Mean concentration at traffic site[c]		Mean concentration at background site[c]		Ratio traffic/ background	
		Traffic site	Background sites	PM	BCP		PM	BCP	PM	BCP	PM	BCP
Janssen, 2008	Munich, Germany March–Dec 2002	Along highway (30.000 veh/ day); 40 m away from another highway (24.000 veh/day)	Suburban residential area	$PM_{2.5}$	Abs. of $PM_{2.5}$ filters;	Annual avg, calculated from 16 week samples per site (adj. for temp variation at ref site)	15.8	2.60	12.2	1.36	1.30	1.91
Boogaard, 2010	8 traffic sites; 9-15 m of busy road in 5 different large cities in the Netherlands; june 2008 – January 2009	Amsterdam; 15.253 veh/24h	Urban background site in the same city	$PM_{2.5}$	Abs of $PM_{2.5}$ filter	Six one week measurements Per site; traffic and corresponding background site measured simultaneously	17.8	4.1	14.8	2.0	**1.2**	**2.1**
		Amsterdam; 9.774 veh/24h					15.1	2.7	15.1	1.6	**1.0**	**1.7**
		The Hague; 17.438 v/24h; canyon					19.4	4.4	16.2	1.6	**1.2**	**2.7**
		Den Bosch; 17.896 v/24h; canyon					18.0	3.7	13.8	1.8	**1.3**	**2.1**
		Den Bosch; 17.138 veh/24h					17.4	2.8	14.5	1.5	**1.2**	**1.9**
		Tilburg; 18.812 veh/24h					17.2	2.4	15.6	1.6	**1.1**	**1.5**
		Utrecht; 13.553 veh/24h					15.4	2.1	14.0	1.4	**1.1**	**1.5**
		Utrecht; 14.831 veh/24h					16.6	3.4	15.1	1.5	**1.1**	**2.2**
Kinney, 2000	New York, USA July, 1996	Busy intersection; 18.375 cars; 2.467 trucks +buses	Control site in quiet residential area	$PM_{2.5}$ mass	EC	8 h (10:00-18:00); 5 obs per site; All sites measured simultaneously	45.7	6.2	38.7	1.5	1.18	4.13
		Bus depot					47.1	3.7	38.7	1.5	1.22	2.47
		Busy intersection; 14.229 cars; 927 trucks + buses					36.6	2.3	38.7	1.5	0.95	1.53
Funasaka, 2000[c]	Osaka, Japan / Sampling period not specified	Outside 5 homes; <5 m from the road; 27.000-29.000 veh/day	Outside homes 60-150 m from the same roads	PM_2	EC	7 days; cascade impactors; area B	27	10	21	6.4	1.29	1.56
						AND sampler; 48-72 h; average of area A+B (7 homes; 25 obs)	30	9.3	24	5..7	1.25	1.62
Roosli, 2001	Basel, Switzerland April 1998 – March 1999	Street canyon near traffic light 18.000 veh/day	Urban background	PM_{10}	EC	Annual average; filters every 4th day analysed	29.9	5.4	21.1	3.0	1.40	1.80
Cyrys, 2003	Munich / March 1999-July 2000	6 sites; average 10 m from traffic	6 sites; urban background	$PM_{2.5}$	EC and Abs. of $PM_{2.5}$ filters;	Annual average, based on 4 2-week samples per site; adjusted for temporal variation at reference site	14.3	3.1	13.3	2.1	**1.08**	**1.43**
	Netherlands; Mar 1999-April 2000	4 sites, average 6 m from traffic	4 sites; urban background				19.9	3.9	17.8	2.1	**1.12**	**1.84**
	Stockholm / Feb 1999-Mar 2000	2 sites, average 19 m from traffic	7 sites; urban background				13.8	2.5	10.2	1.4	**1.35**	**1.79**
Riediker, 2003	Raleigh, USA / Aug-Oct 2001	Near major routes; rotating locations	Fixed ambient site	$PM_{2.5}$	EC	Workshift; 7-9 h; 3pm to midnight; 25 days	29.9	4.0	31.7	1.7	0.94	2.35
Harrison, 2004	London+Birming-ham, UK / April 2000 – Jan, 2002	4 roadside locations; <1 m of kerbside; 27.300-140.400 veh/day	4 background locations; paired to roadside	$PM_{2.5}$	EC	24h; 97 complete sets	22.3	8.4	14.4	2.2	1.6	3.8
Fromme, 2005[c]	Berlin, Ger-many / Feb –June, 2000	Outside 29-33 apartments 14.000-37.000 cars/day	21-23 apartments 100-900 cars/day	PM_4	EC	Daytime; 7-8h; 1 obs per home	32.0	3.4	23.6	2.8	1.36	1.70

[a] Concentrations in $\mu g/m^3$ for PM, black smoke and EC; concentration in $m^{-1}x10^{-5}$ for Absorbance;

[b]: Table only includes results of the 4 schools that were measured in both periods; EC derived from regression equation from 47 co-located EC measurements in 1997/98

[c]: Not specified if samples were conducted simultaneously at traffic and background homes (in grey print)

Table G2. Estimated percentage EC in the roadside increment of $PM_{2.5}$

Reference	Location / period	Measurement method BCP	Difference traffic – background (µg/m^3) PM	Difference traffic – background (µg/m^3) BCP	% EC in roadside increment		
Kinney, 2000	New York, USA; 1996 ; sidewalk	EC; sunset	4.4	2.6		58	
Funasaka, 2000	Osaka, Japan / outside homes; period NA	EC	6.0	3.6		60	
Janssen, 2001; 2008	Netherlands; 1997/98; 50 m of highway	EC from Abs[a]; VDI 2465	2.1	2.0		76 [b]	
Lena, 2002	New York, USA; 1999; sidewalk	EC from Abs[a]; sunset	6.2	3.1		50	
Cyrys, 2003	Munich; 1999/ 2000	EC; VDI 2465	1.0	1.0		80 [b]	
	Netherlands;1999/ 2000	EC; VDI 2465	2.1	1.8		69 [b]	
	Sweden; 1999 / 2000	EC; VDI 2465	3.6	1.1		24 [b]	
Harrison, 2004	London+Birmingham, UK; 2000/02; roadside	EC	8.4	6.1		69	
					**10 µg/m^3 BS = 1.1µg/m^3 EC**	**10 µg/m^3 BS = 0.5µg/m^3 EC**	**10 µg/m^3 BS = 1.8µg/m^3 EC**
Janssen, 1997	Arnhem, NL; Oct–Nov 1994; curbside	Black smoke	7.9	28.3	39	18	64
Roorda-Knape, 1998	NL / May-aug 1995; 15-32 m of highways	Black smoke	1.4	5.5	43	20	71
Roemer, 2001	Amsterdam, NL; 1998/99; 7 m of busy street	Black smoke	2.0	11.0	61	28	99
Fischer, 2000	Amsterdam, Netherlands / 1995; outside homes	Abs of $PM_{2.5}$ filters[c]	3.0	1.3	47	22	77
Smargiassi, 2005	Montreal, Canada; curbside or on collector	Abs of $PM_{2.5}$ filters[c]	1.8	0.7	42	19	68
Janssen, 2008	Netherlands; 2001/02; 50 m of highway	Abs of $PM_{2.5}$ filters[c]	2.4	0.9	43	19	70
Janssen, 2008	Munich, Germany ; 2002; along highway	Abs of $PM_{2.5}$ filters[c]	3.6	1.2	38	17	62
Boogaard, 2010	NL; 2008/09; 9-15 m of busy roads in large cities	Abs of $PM_{2.5}$ filters[c]	2.2	1.6	77	35	127
				**Average[d]**	**55%**	**41%**	**70%**
				**95% CI**	**46-63**	**29-54**	**59-82**

[a] calculated using a study specific calibration derived from co-located samples (see table A1)
[b] Results from studies that have used the VDI protocol were divided by 1.25, as this method has been shown to overestimate EC by on average 25% (Schmid et al, 2001)
[c] An increase in 1 unit of Abs is considered to equal an increase of 10 µg/m^3 BS, according to Roorda-Knape et al. 1998.
[d] Average includes all studies; average of studies that directly measured EC was 61%.

References

Adams HS, Nieuwenhuijsen MJ, Colvile RN, Older MJ, Kendall M. 2002. Assessment of road users' elemental carbon personal exposure levels, London, UK. Atmospheric Environment 36:5335-5342.

Analitis A, Katsouyanni K, Dimakopoulou K, et al. 2006. Short-term effects of ambient particles on cardiovascular and respiratory mortality. Epidemiology 17:230233.

Anderson HR, Bremner SA, Atkinson RW, Harrison RM, Walters S. 2001. Particulate matter and daily mortality and hospital admissions in the west midlands conurbation of the United Kingdom: associations with fine and coarse particles, black smoke and sulphate. Occup Environ Med 58:504-510.

Atkinson RW, Anderson HR, Strachan DP, Bland JM, Bremner SA, Ponce de Leon A. 1999a. Short-term associations between outdoor air pollution and visits to accidents and emergency departments in London for respiratory complaints. Eur Respir J 13:257-265.

Atkinson RW, Bremner SA, Anderson HR, Strachan DP, Bland JM, de Leon AP. 1999b. Short-term associations between emergency hospital admissions for respiratory and cardiovascular disease and outdoor air pollution in London. Arch Environ Health 54:398-411.

Atkinson RW, Anderson HR, Sunyer J, et al. 2001. Acute effects of particulate air pollution on respiratory admissions. Am J Respi Crit Care Med 2001;164:1860-1866.

Behndig AF, Mudway IS, Brown JL, Stenfors N, Helleday R, Duggan ST, et al. 2006. Airway antioxidant and inflammatory responses to diesel exhaust exposure in healthy humans. Eur Respir J 27:359-365.

Boogaard H, Kos GPA, Weijers E, Janssen NAH, Fischer PH, van der Zee S, et al. 2011. Contrast in air pollution components between major streets and background locations: particulate matter mass, black carbon, elemental composition, nitrogen oxide and ultrafine particle number. Atmospheric Environment 45:650-658.

Brauer M, Hoek G, van Vliet P, Meliefste K, Fischer PH, Wijga A, et al. 2002. Air pollution from traffic and the development of respiratory infections and asthmatic and allergic symptoms in children. Am J Respir Crit Care Med 166:1092-1098.

Brauer M, Gehring U, Brunekreef B, de Jongste J, Gerritsen J, Rovers M, et al. 2006. Traffic-related air pollution and otitis media. Environ Health Pespect 114:1414-1418.

Brauer M, Hoek G, Smit HA, de Jongste JC, Gerritsen J, Postma DS. 2007. Air pollution and development of asthma, allergy and infections in a birth cohort. Eur J Respir 29:879-888.

Bremner SA, Anderson HR, Atkinson RW, McMichael AJ, Strachan DP, Bland JM, et al. 1999. Short term associations between outdoor air pollution and mortality in London 1992-4. Occup Environ Med 56:237-244.

Cakmak S, Dales RE, Blanco Vida C. 2009a. Components of particulate air pollution and mortality in Chile. Int J Occup Environ Health 15:152-158.

Cakmak S, Dales R, Gultekin T, Vidal CB, Farnendaz M, Rubio MA, et al. 2009b. Components of particulate air pollution and emergency department visits in Chile. Arch Environ Occup Health 64:148-155.

Cancado JED, Saldiva PHN, Pereira LAA, et al. 2006. The impact of sugar cane-burning emissions on the respiratory system of children and the elderly. Environ Health Perspect 115:725-729.

Cyrys J, Heinrich J, Hoek G, Meliefste K, Lewne M, Gehring U, et al. 2003. Comparison between different traffic-related particle indicators: Elemental carbon (EC), $PM_{2.5}$ mass, and absorbance. J Expo Anal Environ Epidemiol 13:134-143.

Dab W, Medina S, Quenel P, et al. 1995. Short term respiratory health effects of ambient air pollution: results of the APHEA project in Paris. J Epidemiol Comm Health 1996;50(Suppl 1):S42-S46.

Edwards JD, Ogren JA, Weiss JE, Charlson RJ. 1983. Particulate air pollutants: A comparison of British “Smoke” with optical absorption coefficient and elemental carbon concentration. Atmospheric Environment 17:2337-2341.

Fischer PH, Hoek G, van Reeuwijk H, Briggs DJ, Lebret E, van Wijnen JH, et al. 2000. Traffic-related differences in outdoor and indoor concentrations of particles and volatile organic compounds in Amsterdam Atmospheric Environment 34:3713-3722.

Fromme H, Lahrz T, Hainsch A, Oddoy A, Piloty M, Ruden H. 2005. Elemental carbon and respirable particulate matter in the indoor air of apartments and nursery schools and ambient air in Berlin (Germany). Inddor Air 15:335-341.

Funasaka K, Miyazaki T, Tsuruho K, Tamura K, Mizuno T, Kuroda K. 2000. Relationship between indoor and outdoor carbonaceous particulates in roadside households. Environ Poll 110:127-134.

Gauderman WJ, Gilliland F, Vora H, Avol E, Stram D, McDonnel R, et al. Association between air pollution and lung function growth in southern california children. 2002. Am J Crit Care Med 166:76-84.

Gauderman WJ, Avol E, Gilliland F, Vora H, Thomas D, Berhane K, et al. 2004. The effects of air pollution on lung development from 10 to 18 years of age. N Engl Med 351:1057-1067.

Gehring U, Cyrys J, Sedlmeir G, Brunekreef B, Bellander T, Fischer P, et al. 2002. Traffic-related air pollution and respiratory health during the First 2 yrs of life. Eur Respir J 19:690-698.

Harrison RM, Jones AM, Lawrence RG. 2004. Major component composition of PM_{10} and $PM_{2.5}$ from roadside and urban background sites. Atmospheric Environment 38:4531-4538.

Hoek G, Brunekreef B, Verhoeff A, van Wijnen J, Fischer P. 2000. Daily mortality and air pollution in the Netherlands. J Air Waste Manage Assoc 50:1380-1389.

Hoek G, Brunekreef B, Fischer P, van Wijnen J. 2001. The association between air pollution and heart failure, arrhythmia, embolism, thrombosis, and other cardiovascular causes of death in a time series study. Epidemiology 12:355-357.

Janssen NAH, van Mansom DFM, van der Jagt K, Harssema H, Hoek G. 1997. Mass concentration and elemental composition of airborne particulate matter at street and background location. Atmospheric Environment 31:1185-1193.

Janssen NAH, van Vliet PHN, Aarts F, Harssema H, Brunekreef B. 2001. Assessment of exposure to traffic related air pollution of children attending schools near motorways. Atmospheric Environment 35:3875-3884.

Janssen NAH, Meliefste K, Fuchs O, Weiland SK, Cassee F, Brunekreef B, Sandstrom T 2008. High and low volume sampling of particulate matter at sites with different traffic profiles in the Netherlands and Germany: Results from the HEPMEAP study. Atmospheric Environment 42:1110-1120.

Katsouyanni K, Touloumi G, Samoli E, et al. 2001. Confounding and effect modification in the short-term effects of ambient particles on total mortality: results from 29 cities within the APHEA2 project. Epidemiology 12:521-531.

Kinney PL, Aggarwal M, Northridge ME, Janssen NA, Shepard P. 2000. Airborne concentrations of PM(2.5) and diesel exhaust particles on Harlem sidewalks: a community-based pilot study. Environ Health Perspect 108:213-218.

Klemm RJ, Lipfert FW, Wyzga RE, Gust C. 2004. Daily mortality and air pollution in Atlanta: two years of data from ARIES. Inhal Tox 16(suppl 1):131-141.

Lena TS, Ochieng V, Carter M, Holguin-Veras J, Kinney PL. 2002. Elemental Carbon and $PM_{2.5}$ levels in an urban community heavily impacted by truck traffic. Environ Health Perspect 110:1009-1015.

Le Tertre A, Medina S, Samoli E, Forsberg B, Michelozzi P, Boumghar A, et al. 2002. Short-term effects of particulate air pollution on cardiovascular disease in eight European cities. J Epidemiol Comm Health 56:773-779.

Mar TF, Norris GA, Koenig JQ, Larson TV. 2000. Associations between air pollution and mortality in Phoenix, 1995-1997. Environ Health Perspect 108:347-353.

Maynard D, Coull BA, Gryparis A, Schwartz J. 2007. Mortality risk associated with short-term exposure to traffic particles and sulfates. Environ Health Perspect 115:751-755.

Metzger KB, Tolbert PE, Klein M, et al. 2004. Ambient Air pollution and cardiovascular emergency department visits. Epidemiology 15:46-56.

Morgenstern V, Zutavern A, Cyrys J, Brockow I, Gehring U, Koletzko S, et al. 2007. Respiratory health and individual estimated exposure to traffic-related air pollutants in a cohort of young children. Occup Environ Med 64:8-16.

Morgenstern V, Zutavern A, Cyrys J, Brockow I, Koletzko S, Kramer U. 2008. Atopic diseases, allergic sensitization, and exposure to traffic-related air pollution in children. Am J Crit Car Med 177:1331-1337.

Ostro B, Feng WY, Broadwin R, Green S, Lipsett M. 2007. The effects of components of fine particulate air pollution on mortality in California: Results from CALFINE. Environ Health Perspect 15:13-19.

Ostro B, Roth L, Malig B, Marty M. 2009. The effects of fine particle components on respiratory hospital admissions in children. Environ Health Perspect 117:475-480.

Peng RD, Bell ML, Geyh AS, McDermott A, Zeger SL, Samet JM, et al. 2009. Emergency admissions for cardiovascular and respiratory diseases and the chemical composition of fine particle air pollution. Environ Health perspect 117:957-963.

Prescott GJ, Cohen GR, Elton RA, et al. 1998. Urban air pollution and cardiopulmonary ill health: a 14,5 year time series study. Occup Envion Med 55:697-704.

Riediker M, Williams R, Devlin R, Griggs T, Bromberg P. 2003. Exposure to particulate matter, volatile organic compounds, and other air pollutants inside patrol cars. Environ Sci Technol. 37:2084-93.

Roemer WH, van Wijnen JH. 2001a. Daily mortality and air pollution along busy streets in Amsterdam, 1987-1998. Epidemiology 12:649-653.

Roemer WH, van Wijnen JH. 2001b. Differences among black smoke, PM(10), and PM(1.0) levels at Urban Measurement Sites. Environ Health Perspect 109:151-4.

Roorda-Knape M, Janssen NAH, de Hartog JJ, van Vliet PHN, Harssema H, Brunekreef B. 1998. Air pollution from traffic in city districts near major motorways. Atmospheric Environment 32:1921-1930.

Sarnat JA, Marmur A, Klein M, Kim E, Russell AG, Sarnat SE, et al. 2008. Fine particle sources and cardiorespiratory morbidity: an application of chemical mass balance and factor analytical source-apportionment methods. Environ Health Perspect 116:459-466.

Schaap M, van der Gon HAC. 2007. On the variability of black smoke and carbonaceous aerosols in the Netherlands. Atmospheric Environment 41:5908-5920.

Schmid H, Laskus L, Abraham HJ, Baltensperger U, Lavanchy V, Bizjak M, et al. 2001. Results of the 'Carbon conference' international aerosol carbon round robin test stage 1. Atmospheric Environment 35:2111-2121.

Smargiassi A, Baldwin M, Pilger C, Dugandzic R, Brauer M. 2005. Small scale spatial variability of particle concentration and traffic levels in Montreal: a pilot study. Sci Tot Environ 338:243-251.

Tolbert PE, Klein M, Metzger KB, et al. 2000. Interim results of the study of particulates and health in Atlanta (SOPHIA). J Expo Anal Environ Epidemiol 10:446-460.

Tolbert PE, Klein M, Peel JL, Sarnat SE, Sarnat JA. 2007. Multipollutant modeling issues in a study of ambient air quality and emergency department visits in Atlanta. J Expo Sci Environ Epidemiol 7:S29-S35.

Verhoeff AP, Hoek G, Schwartz J, van Wijnen JH. 1996. Air pollution and daily mortality in Amsterdam. Epidemiology 1996;7:225-230.

Zanobetti A, Schwartz J. 2006. Air pollution and emergency admissions in Boston, MA. J Epidemiol Commun Health 60:890-895.

Zeghoun A, Czernichow P, Beaudeau P, et al. 2001a. Short-term effects of air pollution on mortality in the cities of Rouen and Le Havre, France, 1990-1995. Arch Environ Health 56:327-335.

Zeghoun A, Eilstein D, Saviuc P, et al. 2001b. Surveillance des effets a court terme de la pollution atmosphérique sur la mortalité en milieu urbain. Résultats d'une étude de faisabilité dans 9 villes francaises. Revue d'Epidemiol et de Sante Publique 49:3-12.
